# Flat-panel conebeam CT in the clinic: history and current state

**DOI:** 10.1117/1.JMI.8.5.052115

**Published:** 2021-10-28

**Authors:** Rebecca Fahrig, David A. Jaffray, Ioannis Sechopoulos, J. Webster Stayman

**Affiliations:** a Innovation, Advanced Therapies, Siemens Healthcare GmbH, Forchheim, Germany; b Friedrich-Alexander Universitat, Department of Computer Science 5, Erlangen, Germany; c MD Anderson Cancer Center, Departments of Radiation Physics and Imaging Physics, Houston, Texas, United States; d Radboud University Medical Center, Department of Medical Imaging, Nijmegen, The Netherlands; e Dutch Expert Center for Screening (LRCB), Nijmegen, The Netherlands; f University of Twente, Technical Medical Center, Enschede, The Netherlands; g Johns Hopkins University, Department of Biomedical Engineering, Baltimore, Maryland, United States

**Keywords:** conebeam CT, flat-panel CT, digital flat panel, C-arm angiography, interventional radiology, breast CT, extremity imaging, image-guided radiation therapy, dental conebeam CT

## Abstract

Research into conebeam CT concepts began as soon as the first clinical single-slice CT scanner was conceived. Early implementations of conebeam CT in the 1980s focused on high-contrast applications where concurrent high resolution (<200  μm), for visualization of small contrast-filled vessels, bones, or teeth, was an imaging requirement that could not be met by the contemporaneous CT scanners. However, the use of nonlinear imagers, e.g., x-ray image intensifiers, limited the clinical utility of the earliest diagnostic conebeam CT systems. The development of consumer-electronics large-area displays provided a technical foundation that was leveraged in the 1990s to first produce large-area digital x-ray detectors for use in radiography and then compact flat panels suitable for high-resolution and high-frame-rate conebeam CT. In this review, we show the concurrent evolution of digital flat panel (DFP) technology and clinical conebeam CT. We give a brief summary of conebeam CT reconstruction, followed by a brief review of the correction approaches for DFP-specific artifacts. The historical development and current status of flat-panel conebeam CT in four clinical areas—breast, fixed C-arm, image-guided radiation therapy, and extremity/head—is presented. Advances in DFP technology over the past two decades have led to improved visualization of high-contrast, high-resolution clinical tasks, and image quality now approaches the soft-tissue contrast resolution that is the standard in clinical CT. Future technical developments in DFPs will enable an even broader range of clinical applications; research in the arena of flat-panel CT shows no signs of slowing down.

## 1 From Conebeam CT to Flat-Panel CT

The dynamic spatial reconstructor (Mayo Clinic, 1980 to 1990^1^^–^^3^) was one of the first clinical attempts to reconstruct a volume of three-dimensional (3D) data from large-area projection x-ray images. This ambitious vision involved obtaining real-time 3D reconstructions of contrast-enhanced coronary vessels from data acquired in a few milliseconds. Such a feat would push the imaging requirements far beyond what was achievable at the time with state-of-the art single-slice clinical CT scanners. In addition to the need for subsecond imaging of a large volume, this clinical application also required sub-200-μm spatial resolution. The system suffered from a high level of complexity, with multiple x-ray tubes and corresponding x-ray image intensifiers (XRIIs) acquiring images, whereas the whole gantry rotated through partial arcs in ∼140  ms. The XRII itself had several enabling capabilities, including a large area, high resolution, and high frame rates (60 fps and above). Unfortunately, the advantages of the XRII were offset by significant limitations, including the spatial and signal nonlinearities inherent in the electron acceleration technology, in addition to a limited dynamic range (somewhat offset by multiple gain factors) and optical scatter.

Ultimately, the success of the system was hampered by the size and complexity of the system and by the nonidealities of the imaging chain. These same challenges were faced throughout the early- to-mid 1990s by innovators who explored conebeam CT opportunities using XRIIs mounted on radiation therapy simulators,^4^^,^^5^ CT gantries,^6^^,^^7^ dedicated dental scanners,^8^ and angiographic C-arms.^9^^–^^13^

Dynamic, flat-panel, solid-state, x-ray image detectors (DPFs) used in digital fluoroscopy and radiography emerged in the late 1990s. This new generation of dynamic detectors uses a thin layer of x-ray absorptive material deposited on either (1) an electronic active-matrix array fabricated in a film of hydrogenated amorphous silicon (a-Si:H) or (2) an array of active elements integrated on a complementary metal–oxide–semiconductor (CMOS) panel. The nature of the x-ray absorptive material determines if a detector is one of two categories: indirect-conversion (x-ray scintillator-based) or direct-conversion (x-ray photoconductor-based) (see Fig. 6 for an interesting evaluation of commercial flat panels and impact of converter material on modulation transfer function and detective quantum efficiency). As compared with XRIIs, DFPs have no geometrical distortion or vignetting, immunity from blooming in the periphery of high-exposure regions and negligible contrast loss due to internal scatter. DFPs exhibit a wider dynamic range, as compared with (medical imaging) XRIIs, which have limited dynamic range within each electronic gain stage. DFPs provide high resolution over a large area, whereas XRIIs can do so only for small fields of view. Although the earliest flat panels suffered from high electronic noise, by the mid-2000s, the detective quantum efficiency of flat panels exceeded that of image intensifier systems for all but the lowest doses (i.e., <1  μR per frame at the detector).^14^^,^^15^ Finally, manufacturing costs have dropped significantly over the two decades since the introduction of DFPs, and many fabrication facilities can now easily switch their production lines between flat panels for medical imaging and for consumer electronics.

**Fig. 1:**
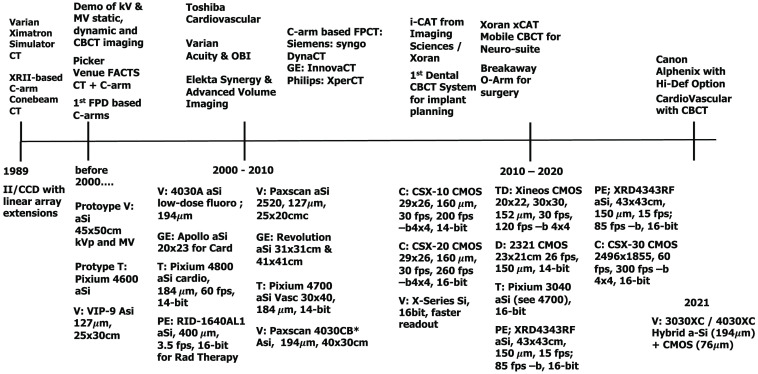
Incomplete but indicative timeline of the development of DFP technology over the last two decades. The evolution in detector size, frame rate, pixel size, and bit-depth is captured. Manufacturers of both aSi:H and CMOS first provided small(er) panels suitable for cardiology and have then evolved their technology to cover large areas, faster readout rates, and higher bit depth. All product names should be considered registered trademarks of the respective companies. V, Varian; GE, General Electric; C, Canon; D, Dexela; T, Trixell; TD, Teledyne DALSA; PE, Perkin Elmer; -b, binned; *dual gain and dynamic gain switching modes.

DFPs have replaced XRIIs for fluoroscopy-guided diagnosis and intervention and have been integrated into on-line patient position verification systems in radiation therapy using alternative, higher-absorptivity conversion materials for use with MV photons. They have also enabled dedicated conebeam CT imaging systems for breast, dental, head, and extremity imaging. An indicative (and incomplete) timeline highlighting technical developments of digital flat panels (DFPs) is presented in Fig. 1. Development of new converter-material combinations, new readout technologies, and new direct-conversion technologies has led to lower noise floors, higher frame rates, and expanded dynamic range, all while maintaining the large-area format and high resolution.

The flat-panel detector allows for wide z-axis coverage, resulting in the ability to image entire organs, such as the brain, heart, liver, breast, or kidneys, in a single axial rotational acquisition. In addition, the detector provides ultrahigh spatial resolution in both the radial and axial directions. In general, flat-panel CTs (FPCTs) have continued to focus on clinical applications that require a combined very high spatial and contrast resolution, such that the dose-to-voxel volume trade-off is deemed acceptable in the context of the ALARA principle. While early DFP technology did not meet all of the imaging requirements for all clinical applications (especially readout-rate), the geometric fidelity and high resolution of the detectors allowed for the implementation of flat-panel CT for “semistatic” imaging tasks, such as neurovascular imaging; 3D radiation oncology treatment verification; 3D mammographic CT; and extremity, dental, and ear, nose, and throat (ENT) imaging.

In this historical and technical review, we present the development of flat-panel CT with a focus on four clinical applications: breast FPCT, fixed C-arm FPCT, on-board FPCT, and extremity/head FPCT. Table 1 summarizes parameters for current “state-of-the-art” systems that are available today. This is a single snap-shot in time and as the continuous development of DF detector technology in Fig. 1 demonstrates, we expect further improvements in capabilities and in the resulting image quality for an ever-expanding range of imaging applications.

**Table 1:** Summary of system parameters for the clinical applications described in this historical review.

	Detector specifications	System geo.	Acq. geometry	Image chain specs.	Notes
Breast CT	MAT: 1024 × 655–b PS: 384 FP: 40 × 30 FR: 30 TFT	SDD: 92.3 Mag: 1.4	Proj: 300 to 500 AR: 360 ST: 10 to 16	PW: ∼10 kV: 49 to 65 FIL: 1.6 mm Al to 0.2 mm Cu FS: 0.1 to 0.3	
	*or* MAT: 1944 × 1536* PS: 150 FP: 29 × 23 FR: 30 CMOS	SDD: 69.9 Mag: 1.4			
Fixed C-arm CT	MAT: 1024 × 1024 MAT: 1240 × 960 and smaller PS: 100 to 200* FP: 20 × 20, 40 × 40 FR: 15, 30, 60	SDD: ∼120 Mag: 1.4 to 1.7	Proj: 130 to 600 AR: 200 to 220 (260) single sweep**; New traj # ST: 3.5 to 20	PW: up to 100 kV: 50 to 125 FIL: Cu FS 0.3 to 1.0	* 75 μm available ** Robotic trajectory with moving center of rotation to increase FOV # wobble/sinusoidal trajectory to reduce conebeam artifact aSi:H and CMOS
Cone-beam CT for image-guided radiotherapy	MAT: 1024 × 1024 –b2 × 2 PS: 200 –b 2 × 2 FP: 41 × 41	SAD: 100* Mag: 1.2 to 1.6	Proj: e.g., 600 AR: 190 to 360 offset detector geometry to increase FOV up to 50 cm in diameter ST: 60**	PW: up to 100 kV: 40 to 130 FIL: Bow-tie + var. apertures FS: 0.5 to 1.2	** Rotation speed limited to 1 RPM for C-arm accelerators Cone-beam CT now also available on circular gantry designs without speed restriction. Support for 4D cone-beam CT. * Allows radiographic simulation of MV beam geometry Both with CsI:Tl
	*or* MAT: 1024 × 768 –b 2 × 2 PS: 194 –b 2 × 2 FP: 40 × 30				
Head	MAT: var. PS: var. FP: 5 × 5 to 25 × 25* FR: var. CMOS and aSi	Variable SDD: 50–70 range Mag: 1.2 to 2	Proj: var, 180 to 360 typical AR: 1890 to 360 ST: 5 to 30	PW: ∼1 typical kV: 60 to 120 FIL: Al 2 to 14 mm FS ∼0.4 to 0.5 VS: 75 to 400 μm	Highly variable Most systems plug into wall power
Extremity	AT: 1500 × 2000 to 1800 × 2200 typical, –b 2 × 2 typical PS: 130 to 300 FP: 25 × 30 FR: 15 to 25	SDD: ∼55 Mag: 1.3 to 1.8	Proj: 200 to 600 AR: 210 to 360 ST: 18 to 60	PW: ∼1 kV: 60 to 120, 80 to 110 typical FIL: Al FS: ∼0.6 VS: 150 to 350 μm	

MAT, matrix; –b, binned; PS, pixel size in μm; FP, flat panel dimensions in cm; VS, voxel size of the 3D reconstructions in μm; Mag, magnification; SDD, source-to-detector distance in cm; SAD, source to axis distance in cm; TFT, thin-film transistor; CMOS, complementary metal–oxide semiconductor; Proj, number of projections; AR, angular range in degrees; ST, scan time in s; FIL, filtration; kV, kilovoltage range; FS, focal spot size in mm.

The systems available in the 2010s had limited detector readout speed, truncation of the trans-axial field of view, limited dynamic range, increased scatter due to large-volume irradiation, and required reconstruction using incomplete data due to the conebeam acquisition geometry. Some of the algorithms developed to mitigate the artifacts arising from the detector limitations are described briefly here. Further developments of the detector technology, including increased frame rates and dynamic range (including dual-gain readout) and improved noise floor (e.g., due to the use of CMOS instead of aSi:H), have enabled “close to true CT” image quality, with low-contrast resolution approaching 5 HU for voxel sizes of ∼150  μm. Nonetheless, all FPCT systems continue to require dedicated correction algorithms. New opportunities, especially driven recently by the development of AI-based algorithms for image processing and reconstruction, are opening interesting avenues for further improvements.

## 2 Reconstruction and Artifact Correction

In the following, knowledge of two-dimensional (2D) CT reconstruction is assumed. An excellent review of CT reconstruction algorithm history is provided in Ref. 16. The artifacts that are common to both traditional and flat-panel CT, such as beam hardening, scatter correction, and spectral correction, are not addressed here since solutions are, in general, similar between standard diagnostic CT and flat-panel-based CT systems.

A literature search in PubMed using the terms [(conebeam CT reconstruction) AND (flat panel)] returns a total of 207 publications between 1999 and 2021, indicating that the topic continues to be an active area of research. In diagnostic flat-panel CT, such as for applications in breast and dental imaging, longer times between image acquisition and reconstructed volume viewing may be acceptable. However, in both radiation therapy and interventional image guidance, image data must be provided during the therapeutic session and therefore fast reconstruction is an absolute necessity. An “acceptable” reconstruction time is <1  min, and a “barely adequate” reconstruction time would be within 3 min. In addition, some level of soft-tissue visualization (e.g., fat/tissue separation) requires that at least 100 (preferably 600 or more) views be acquired.

For these reasons and others, most clinical systems use direct (noniterative) reconstruction with algorithms that permit independent processing of each projection (or of at most two or three acquired in sequence) followed by backprojection in 3D. A few iterations may be used to correct for nonidealities, such as scatter and beam hardening. In general, the standard fanbeam convolution-backprojection algorithms produce unacceptable artifacts for cone angles greater than ∼8  deg,^17^ and therefore reconstruction algorithms targeted to large-cone-angle FPCT were developed.

The following “completeness condition” must be true if an analytic solution to the 3D reconstruction problem is desired: “If on every plane that intersects the object there lies a vertex, then one has complete information about the object.”^18^^,^^19^ Some mechanical motions that meet this condition have the apex of the cone moving on one of the following trajectories: a complete sphere, a hemisphere, two orthogonal circles, a helix, a circle and an orthogonal line, or the pattern made by the stitching on a baseball.^20^^–^^22^ To keep mechanical implementation simple, earliest FPCT systems used a (close to) circular trajectory, and almost exclusively used some version of the Feldkamp–Davis–Kress (FDK) algorithm,^23^ a straightforward (but approximate) extension of fanbeam filtered-backprojection. We start here with a description of the mathematically exact algorithm first described by Grangeat, which can be applied to arbitrary trajectories for which complete data are available.^24^^–^^26^ This algorithm reduces to the FDK algorithm for the standard (incomplete) perfect-circle trajectory still used in many FPCT imaging systems today.

### 2.1 Exact Reconstruction Using Conebeam Backprojection

Inversion of the 2D Radon transform for parallel-beam CT reconstruction using convolution backprojection is described by La Rivière and Crawford.^16^In analogy with the 2D Radon transform, inversion of the 3D Radon transform is also possible. This property of the Radon transform was exploited by Grangeat in his description of an exact reconstruction algorithm first published in 1987.^24^

**Fig. 2:**
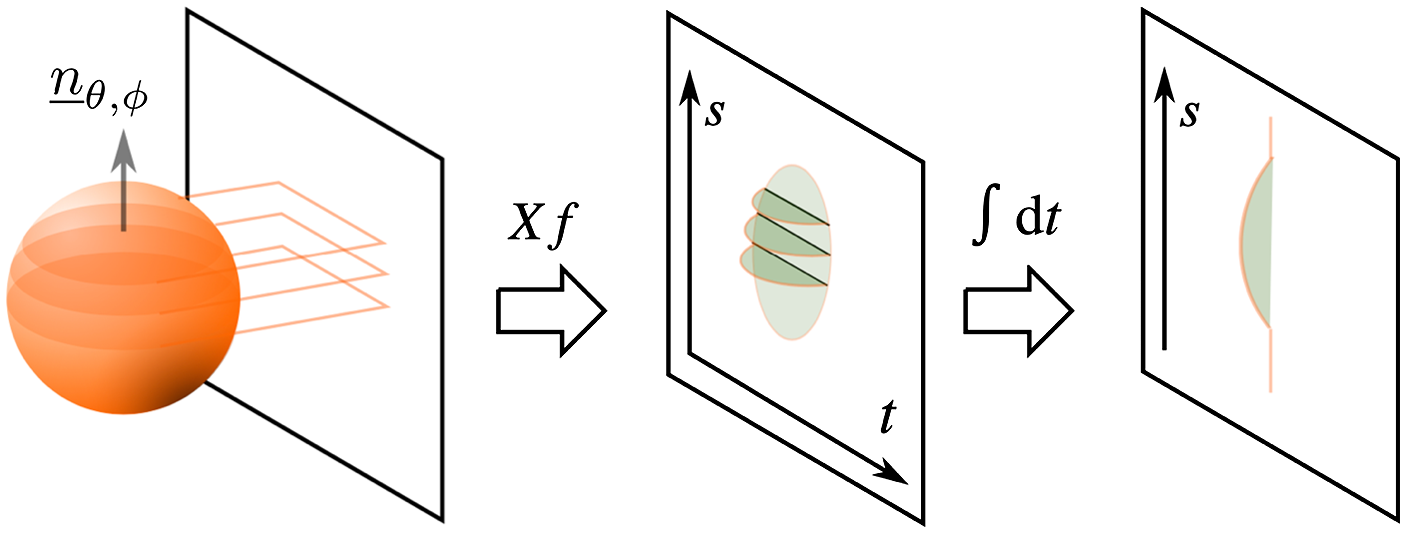
Modified from Ref. 27 under the terms of the Creative Commons Attribution 3.0 License. Schematic showing the relationship between the 2D x-ray projection and the 3D Radon transform. The conventional x-ray projection image is formed by the integral through the object along the direction perpendicular to the detector. By integrating along a direction parallel to the detector (denoted by t in the following derivation), an integral over one plane through the object is performed and hence provides the prerequisite data for the 3D Radon transform.

To understand this approach, we link the 2D Radon transform, the 3D Radon transform, and the x-ray transform. The x-ray transform and the 2D Radon transform are identical in the 2D case since an n−D construct is projected onto an (n−1)-D dimensional construct, i.e., a plane is projected onto line integrals. In 3D, this is different as Fig. 2 shows. The 3D x-ray transform projects the object’s information onto one-dimensional integrals, i.e., line integrals. The 3D Radon transform, however, integrates the 3D object onto 2D plane integrals. Hence, the functions are different, yet related. In fact, part of the 3D Radon transform can be determined from 2D x-ray views, by computing line integrals of the projection (see Fig. 2). Each line on the detector forms a plane with the respective x-ray source. Therefore, computing the line integral on the pixelwise x-ray line integrals will form a plane integral. As Grangeat showed, this is also true, up to a weighting for cone-beam geometries.^24^^–^^26^

Based on these observations, Defrise and Clack have proposed an algorithm that can be applied to a multitude of continuous acquisition trajectories, yet reduces to the FDK-method for circular scans; it is presented here for a flat-panel geometry.^28^

**Fig. 3:**
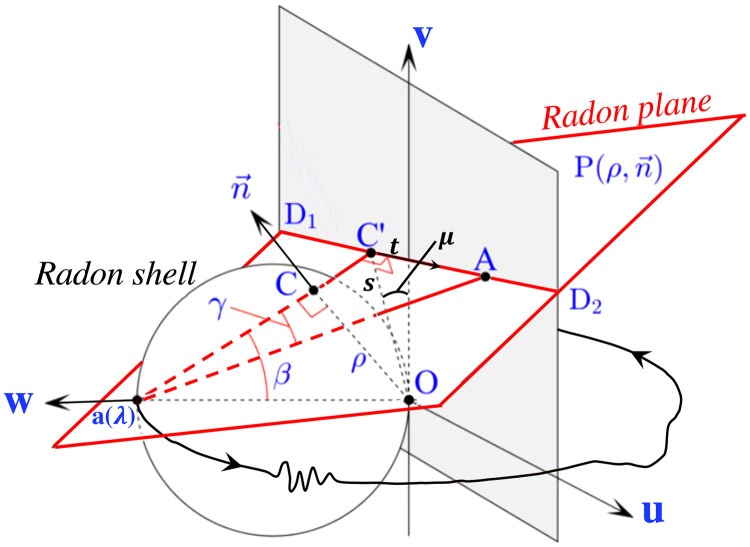
Schematic showing the geometry of FPCT data acquisition by cone shaped x-ray beam and a flat panel detector, and the relationship among detector, x-ray transform (e.g., the line integral along a(λ)A), Radon plane, and Radon shell. The focal spot trajectory is affected by bearing jitter and gantry sag, causing deviations from a perfect circular trajectory. See Ref. 29 for a standard illustration.

Since each cone-beam projection is filtered independently, we define for each focal spot at location λ a 3D coordinate system attached to the detector, such that the origin O is the orthogonal projection of the orbit point a(λ) onto the detector plane. Orthonormal vectors u and v are chosen within the detector plane (see Fig. 3).

Referring to Fig. 3, we describe in general terms the outline of the algorithm, which can be clustered into the main steps of cosine-weighting, filtering, and cone-beam backprojection, as is typical for fanbeam reconstruction in CT.

Step 1: Cosine weighting of cone-beam projection (u,v,λ), where u and v are the 2D detector coordinates, projection angle λ, and source–detector distance D distance between a(λ) and O]:
  $$p_{1}(u,v,\lambda )=\frac{D}{\sqrt{u^{2}+v^{2}+D^{2}}}p(u,v,\lambda ).$$
Step 2: Radon-based filtering (equivalent to rowwise ramp filter for circular orbits).
Step 2.1: Compute the 2D parallel Radon transform (cf. Fig. 3), where s is the perpendicular distance from the origin to the virtual detector row (corresponding to the intersection of the Radon plane with the detector) and μ is the angle of rotation of the virtual detector row. Integrate over t=−∞ to ∞, or equivalently from D1 to D2 for a finite-sized detector:
  $$R_{1}(s,\mu ,\lambda )=R\{f_{1}(u,v,\lambda )\}=\int_{t=-\infty \;\text{to}\;\infty }p_{1}(s\;\cos\;\mu +t\;\sin\;\mu ,s\;\sin\;\mu -t\;\cos\;\mu ,\lambda )dt.$$
Step 2.2: Differentiate with respect to s
  $$R_{2}(s,\mu ,\lambda )=\frac{\partial R_{1}(s,\mu ,\lambda )}{\partial s}.$$
Step 2.3: Multiply with the trajectory-dependent weighting factor M(s,μ,λ)
  $$R_{3}(s,\mu ,\lambda )=-\frac{1}{4\pi^{2}}M(s,\mu ,\lambda )\cdot |\cos\;\mu |\cdot R_{2}(s,\mu ,\lambda ),$$
  with
  $$M(s,\mu ,\lambda )=\frac{{\left|\cos\;\mu \right|}^{m}}{2|\cos\;\mu {|}^{m}+2\;\max{\left(1-{\left(\frac{s}{D}\;\cos\;\lambda -\cos\;\mu \;\sin\lambda \right)}^{2},0\right)}^{m/2}},$$
  and m>2. Here, max (a,b) will return a if a>b and b if b>a. A common choice is m=4.^24^
  Note that for s, μ, and λ, the actual parameters of the trajectory are required.
Step 2.4: Differentiate again with respect to s
  $$R_{4}(s,\mu ,\lambda )=\frac{\partial R_{3}(s,\mu ,\lambda )}{\partial s}.$$
Step 2.5: Backproject into the original projection
  $$p_{2}(u,v,\lambda )=(u^{2}+v^{2}+D^{2})\cdot R^{-1}\{f_{1}(u,v,\lambda )\}=(u^{2}+v^{2}+D^{2})\int_{\mu }{R_{4}(t,\mu ,\lambda )|}_{t=u\;\cos\;\mu +v\;\sin\;\mu }d\mu .$$
Step 3: Backprojection of the cone-beam projections
  $$f(x)=\int_{\lambda }\frac{1}{C^{2}}p_{2}(P(x),\lambda )d\lambda ,$$
  where C=|x−a(λ)| is the distance between the voxel x under consideration and the source position and P(x) is the cone-beam projection of x onto the detector coordinates u and v.

### 2.2 Feldkamp–Davis–Kress for Circular Trajectory

For step 1 of the above algorithm on a circular trajectory, factor $\frac{D}{\sqrt{u^{2}+v^{2}+D^{2}}}$ is applied prior to convolution with an appropriate apodized filter, and the factor corrects for the difference in path length (or difference in number of voxels to which a particular value in the projection is applied) for rays detected at the edge of the detector compared to rays detected at the center of the detector. The factor $1/C^{2}$, applied to the convolved projections during backprojection, accounts for the dependence of ray density at the point (s,μ) on the distance of the point from the x-ray source. For those systems that acquire 2D projection images over only 180 deg plus the fan angle, Parker’s “short scan” weighting scheme^30^ can be used to smoothly scale the data that are acquired twice, prior to reconstruction.

**Fig. 4:**
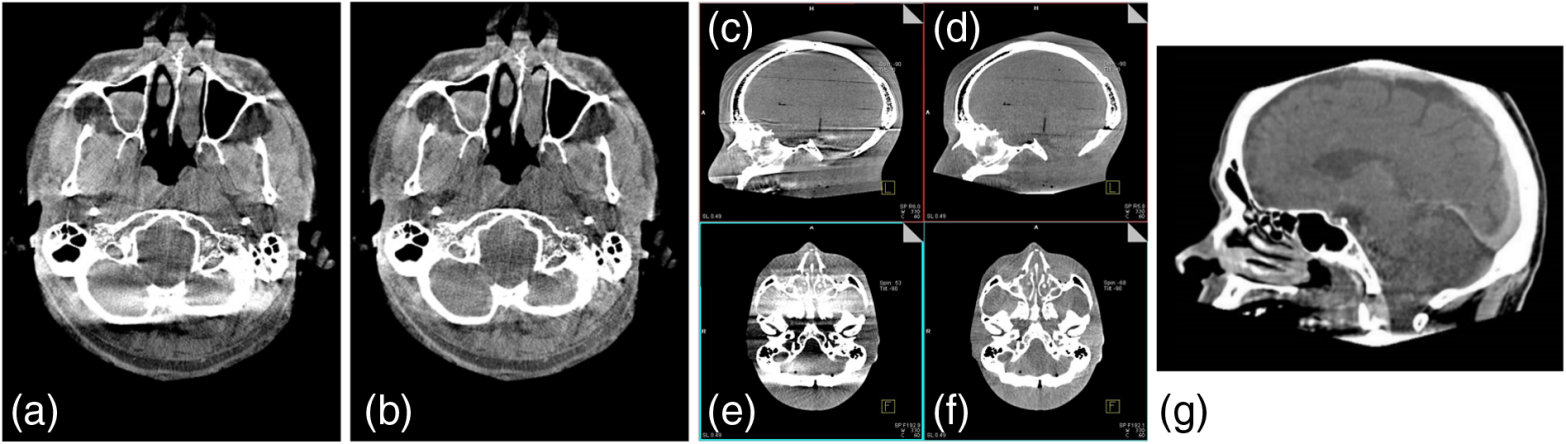
Comparison of reconstruction algorithms for FPCT reconstructions of the head. (a) FDK-type reconstruction; (b) same data reconstructed using a Grangeat-type reconstruction algorithm; (c) and (e) coronal and axial slices through a volume reconstruction of a phantom for data acquired on a “mostly circular” trajectory (circular but with deviations due to gantry sag, bearing jitter, and other C-arm gantry nonidealities), Grangeat-type reconstruction; (d) and (f) coronal and axial slices through a volume reconstruction of a phantom for data acquired using syngo DynaCT Sine Spin™ (a sinusoidal trajectory) showing improved soft tissue image quality especially in the posterior fossa and skull base; (g) clinical reconstruction using the same acquisition geometry and reconstruction as for (d) and (f).

A comparison of reconstruction image quality for a circular trajectory acquisition reconstructed using FDK and using an exact reconstruction based on Grangeat’s work is shown in Fig. 4. An extension of the circular trajectory by including x-ray source motion in a smooth sinusoidal pattern above and below the plane, reconstructed using Grangeat’s work, is also shown. This trajectory has been proposed as a universal coupled source–detector trajectory for reducing artifact from high-contrast regions.^31^

### 2.3 Iterative Reconstruction and Deep-Learning

Although iterative algebraic reconstruction was the first approach used to reconstruct tomographic data from x-ray projections, it was quickly supplanted by the more computationally efficient (from both speed and data access requirements) filtered backprojection algorithms. As CPU and GPU hardware have continued to evolve and associated computation power has increased, investigations into the value of iterative approaches have resurfaced. In iterative reconstruction, the image acquisition/reconstruction process is modeled as a system of linear equations with the number of observations (x-ray line projection measurements) usually far exceeding the number of voxels in the volume to be reconstructed. Given the discretized version of the x-ray projection,

Ax=p,

where A is a system matrix that describes the projection of a linearized volume x to a linearized vector p that contains all observed detector measurements. The above system of equations can be solved using Radon’s ideas to yield

$$x=(A^{T}A)+A^{T}p,$$

where $A^{T}$ is a discrete version of the backprojection operation and ${\left(A^{T}A\right)}^{+}$ is a circulant Moore–Penrose matrix that takes the role of a filtering step. In general, the system of equations represented by the above is quite large, making the matrix inversion too computationally expensive for direct evaluation. Instead, an iterative approach is typically adopted, successively minimizing an error term that compares the measurements with simulated projections. This formulation is flexible and can be used both for overdetermined systems (more measurements than voxels) and underdetermined systems (e.g., sparse data acquisitions) and may include regularization terms that include *a priori* knowledge to improve the conditioning of the problem, control noise, and enforce desirable image properties in the reconstructed image. The challenge with iterative approaches continues to be the need for instance-specific tuning of several control parameters, which have a significant impact on trade-offs between spatial resolution and noise in the “final” reconstruction. Interestingly, first FPCT-based products that use iterative reconstruction have recently been brought to the market, e.g., iCBCT, currently a standard offering on Varian’s Halcyon™ and Ethos™ platforms.

Research combining iterative reconstruction and correction approaches with AI/deep learning has exploded in the last 5 years, indicating that AI-based iterative reconstruction may become mainstream again.^32^ Pure machine learning methods suffer from the problem that they will only be applicable to observations that match the training distribution. The wide range of variation in medical imaging and human anatomy poses a challenge to postprocessing and purely data-driven methods. Using the above matrix notation for image formation and reconstruction, individual operations can be mapped onto a neural network. For example, you can have a parameterized solution of the following form:

$$x=A^{T}\mathrm{FCp}.$$

**Fig. 5:**
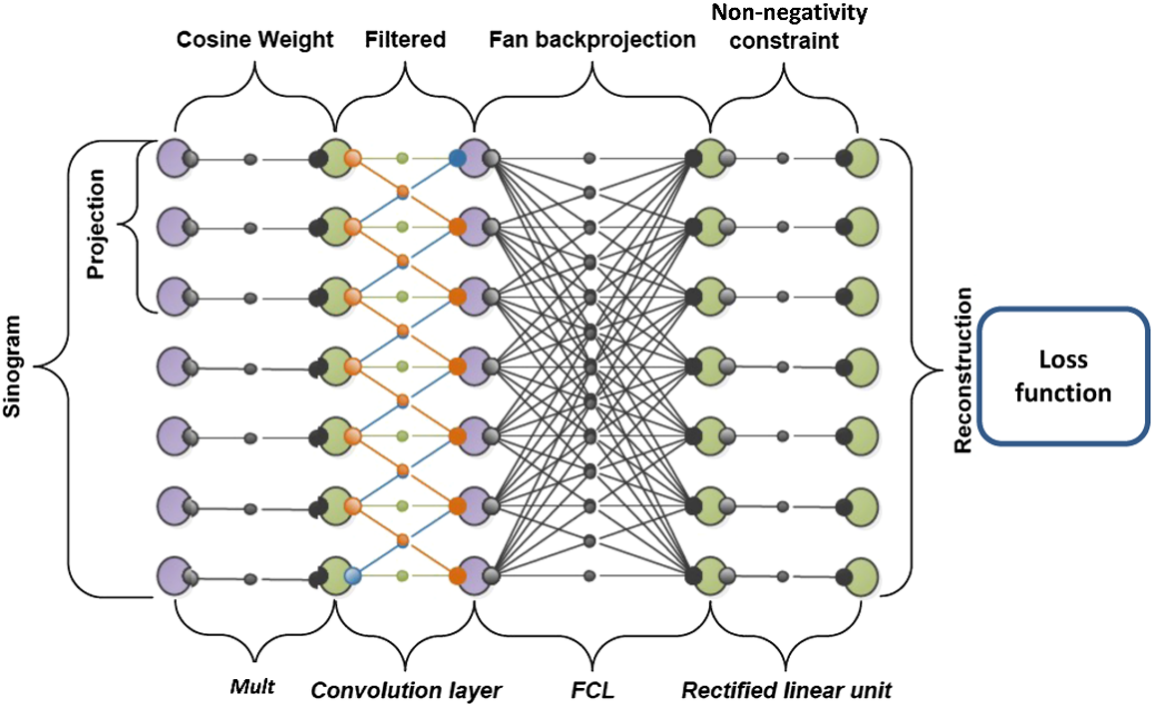
Visual representation of the matrix inversion equation as a neural network.

For example, C could be an optimized trainable projection-domain weighting with (more optimal) redundancy weights, and F could be a learned filtering optimization that applies to a specific acquisition geometry (i.e., limited angle) while maintaining the known physics and geometry of backprojection in the system matrix A. Similarly, fan-beam limited-angle reconstruction can be tackled by encoding 3D geometries in A.^33^ A neural-net representation of the equation is shown in Fig. 5. Radon-based filtering as in the Defrise–Clack algorithm could also be integrated into such hybrid deep neural networks. In contrast to completely data-driven approaches, these hybrids come with the guarantee of a reduced error in terms of maximal error bounds and increased generalization ability.^34^ In other approaches, deep learning has also been used to reduce cone-angle artifact in circular cone-beam CT^35^ and to generate an image-based geometry correction.^36^

### 2.4 FP-Related Artifact Management in FPCT

A recent review article by Tang et al.^29^ provides an excellent review of cone beam artifacts and methods to correct for those artifacts in axial conebeam CT. We therefore provide a historical view of earlier work done to mitigate those artifacts that arise specifically due to use of a DFP, covering lag and dynamic range, and truncation in the trans-axial direction.

*a-Si:H.* The amorphous silicon flat panel detector uses indirect technology, with a scintillator layer (often columnar CsI:Tl) that converts x-ray photons to light, and a second layer of photodiodes and active-matrix thin-film transistors made in amorphous silicon. The photodiodes convert photons to electrons and the transistors switch out the current that is collected on the capacitance of the diodes.

*Complementary Metal Oxide Semiconductor (CMOS).* CMOS DFPs also use a scintillator such as CsI:Tl to convert incident x-rays into visible photons and a photodiode to convert visible photons into electrons for subsequent readout. CMOS technology offers a variety of potential advantages in material characteristics over a-Si:H, including lower dark current (reduced electronic noise), higher charge mobility (lower image lag and higher frame rate), and fabrication with finer pixel pitch compared with a-Si:H active matrix DFPs. New transistor implementations have been developed that mitigate the sensitivity to radiation damage that was seen in early CMOS FPs.

**Fig. 6:**
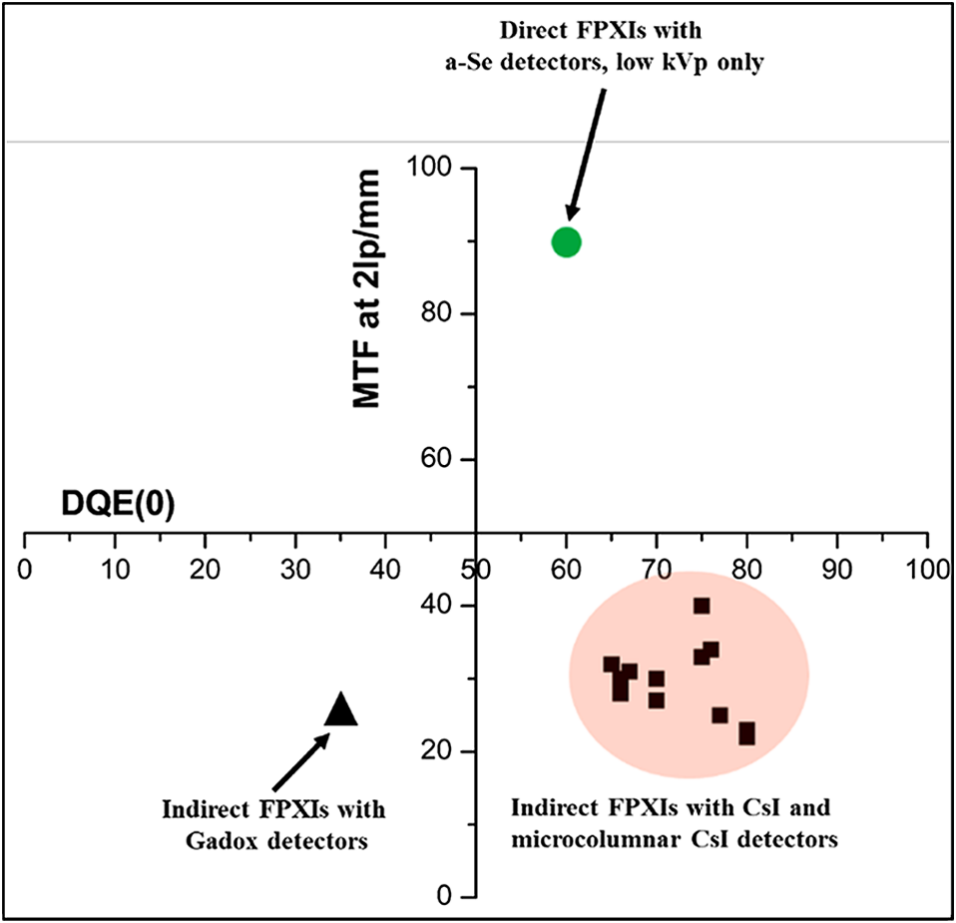
Detective quantum efficiency (DQE) versus modulation transfer function for 17 commercial off the shelf products. The DQE(0) for CsI and Gadox FPs is for a dose of 1  μGy. Modified from Ref. 37.

An interesting visualization of the current status of commercial off-the-shelf flat panels is shown in Fig. 6. Both CMOS and a-Si:H panels fall into the lower right quandrant (especially when comparing the 2  lp/mm point on the curve) since they use similar scintillator converter technology. In spite of the attractive noise performance of CMOS FPs, a-Si:H panels continue to be more widely used due to CMOS panel size limitations and cost. An extensive body of work describing modeling, measurement, and calibration techniques to correct for the nonidealities of a-Si:H panels exists. A pictorial summary of the image-correction process implemented for a typical a-Si:H detector is shown in Fig. 8.

*Correcting for lag.* The use of amorphous silicon leads to slow discharge rates of the transistor elements, and therefore some signal accumulated in one frame is read out in subsequent frames. A second source of lag is scintillator afterglow, although this effect is much smaller than the former. A PubMed search on “flat panel lag” produces 39 results for the time 1999 to 2021.

Both software^38^^,^^39^ and hardware methods to deal with lag in flat-panel imagers (FPIs) were developed early on. One effective hardware method used LEDs built into the panels to backlight the photodiodes, which saturates the traps.^40^ Forward biasing to push current through the diode can be used to achieve the same goal.^40^^–^^42^ These hardware approaches do not correct for scintillator afterglow.

In software correction, an impulse response function (IRF) is generated by fitting a suitable model, such as a multiexponential^43^ or power function,^44^ to the lag decay. A single IRF can be used to describe the entire panel, or an independent function can be fit for each individual pixel, which allows for variation in lag across the detector. A basic assumption of the IRF model is that the panel acts in a linear and time-invariant way. The correction is then provided by a temporal deconvolution of the detector output with the modeled IRF. The accuracy of the linear, time-invariant model depends on the size of the object (i.e., on the dynamic range seen by individual pixels) and on the exposure level at which the calibration of lag coefficients and lag rates is carried out. This implies that large signal variations across the panel, such as those near the boundary of an object, show nonlinear behavior.^45^^,^^46^

Implementation of software algorithms for lag correction continues to be an area of research, although most industry users of a-Si:H technology have chosen to implement corrections in reconstructed image space rather than implement a lengthy system-specific calibration process (see Fig. 8).

**Fig. 7:**
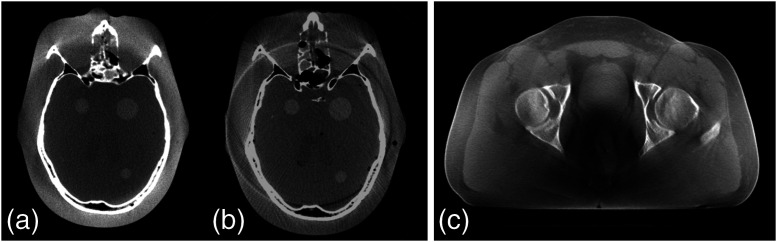
(a) FPCT reconstruction of a head phantom using the dual-gain mode of a Paxscan 4030CB 16-bit flat panel; (b) the same phantom imaged using a 14-bit flat panel—note increase in streaks and ring artifacts. (c) FPCT reconstruction of a human pelvis also using the dual-gain mode. Prior to reconstruction, the projection images were processed using a severe unsharp masking algorithm to enhance any possible boundary artifacts resulting from gain switching—no such boundary artifacts are visible. Images courtesy Ed Shapiro and Rick Colbeth, Varex Imaging.

*Dynamic range limitations.* The first generation of a-Si:H DFPs provided first 12-bit and then 14-bit analog-to-digital converters (ADCs), and CT scanners of the same era already had detectors with 18-bit ADCs.^49^ More recent generations of a-Si:H provide 16-bit standard. The limited bit-depth of the DFP projections caused degradation of conebeam CT image quality: (1) large relative errors in beam intensity values measured through dense anatomy, resulting from too large quantization steps, cause streak artifacts off dense objects, (2) insufficient resolution of pixel gain and offset normalization caused ring artifacts, and (3) truncation of low density anatomical detail at patient boundaries, due to insufficient dynamic range, results in shadow artifacts and incorrect Hounsfield numbers. A comparison of 14-bit versus 16-bit FPCT images is shown in Fig. 7 in addition to an FPCT acquired using a dynamic gain readout amplifier that extends the bit-depth to 16.4, demonstrating excellent reconstruction fidelity out to the body boundary.^50^

*Truncation in the trans-axial direction.* The maximum dimension of a-Si:H detectors has so far been limited to ∼40  cm, leading to truncation in the trans-axial direction for 3D imaging of lung fields, abdomen, and hips (e.g., during surgical pin placement guided using mobile C-arms). For such cases, truncation of projection images leads to severe artifacts around the edges of the reconstructed FOV and also to inaccuracies in the reconstructed HU values due to the associated cupping effect in the image.

**Fig. 8:**
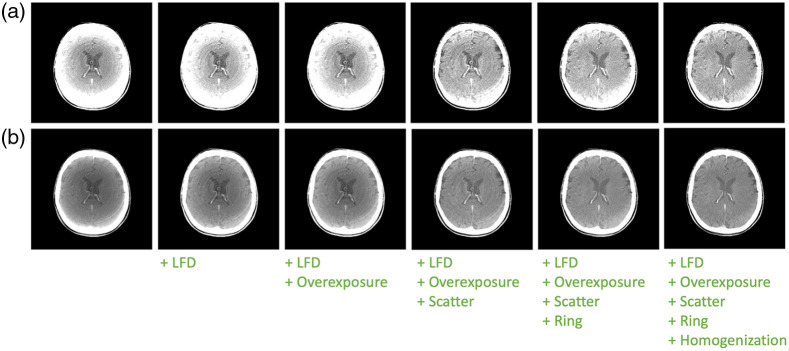
Illustration of the application of standard correction steps to data acquired using a 30×40  cm a-Si:H 16-bit flat panel. Some corrections such as “overexposure” and “truncation” have only a minor effect on this rather small volume. Original data courtesy of Drs. Arnd Doerfler, Stefan Lang, and Philip Hoelter, University Hospital Erlangen; corrections courtesy Dr. Michael Manhart. All images include standard I0, log, and water correction. (a) Window width 100. (b) Window width 200. + indicates application of a correction for the listed nonideality. LFD, low-frequency drop.^47^^,^^48^

Building on early work in diagnostic CT imaging, several different techniques were proposed to estimate the missing data based on either *a priori* knowledge about the data or using redundancies in the data including: symmetric mirroring,^51^ use of redundant rays,^52^ polynomial extrapolation,^53^^–^^55^ use of *a priori* information,^56^ iterative techniques as well as non-FDK reconstruction approaches such as projection onto PI-lines,^52^ and the truncated Hilbert transform.^57^ In radiation therapy where focal-spot-to-detector distances are even longer and thus trans-axial truncation is even more severe, use of an off-set detector and a full 360-deg acquisition is the standard practice; the same geometry is often used in dental CT, where cost considerations mandate use of as small a detector as possible. Algorithms to correct for sampling and filtering discontinuities in the overlap region were developed (Fig. 8).^58^^–^^61^

## 3 FPCT (kV and MV) in Radiation Therapy

### 3.1 Flat-Panel CT for Image-Guided Radiotherapy

**Fig. 9:**
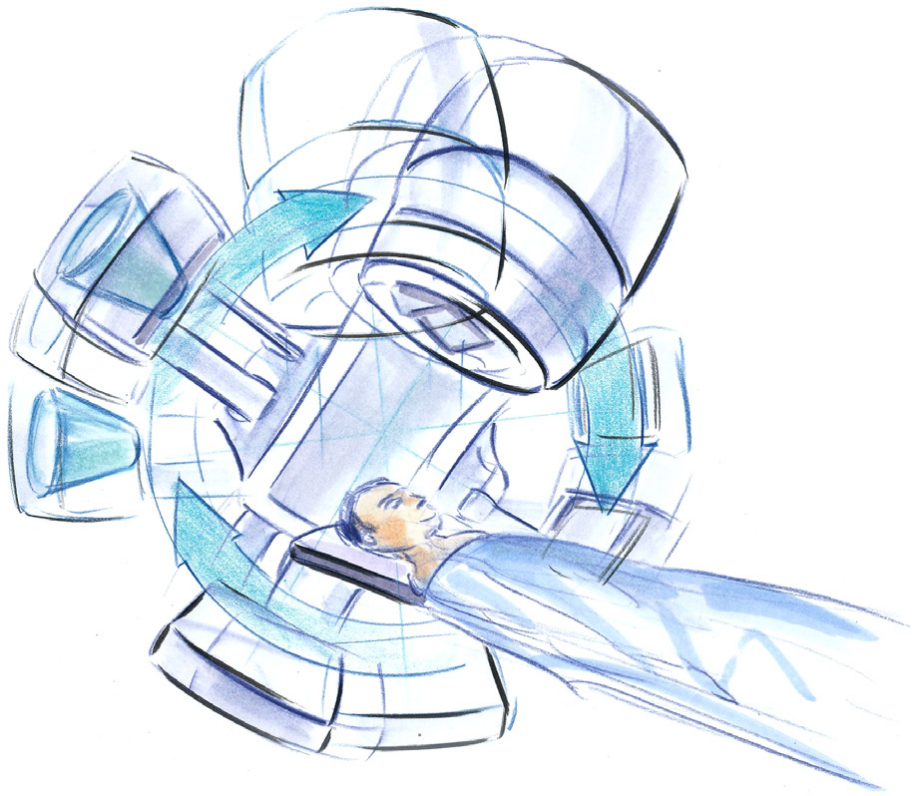
Artist’s rendition of FPCT implemented on a radiation therapy treatment system. The FPCT system is installed on the same gantry as the treatment head, with an offset of 90 deg.

Radiotherapy is an essential component of effective cancer control, and clinical evidence supports its use in over 50% of cancer patients at some point in their care. As a local therapy, it is critical to provide accurate and precise placement of the radiation dose within the body. Failure to cover the desired target tissues will undermine the likelihood of success while covering excess tissues increases the likelihood of normal tissue toxicity. In the 1990s, advances in computing for personalized treatment planning and the development of electromechanical radiation beam shaping devices such as multileaf collimators enabled the creation of highly conformal dose distributions and escalated the need for greater precision and accuracy in dose placement at each treatment fraction.^62^ The traditional use of portal films or portal images based on the megavoltage energy treatment beams did not provide the capacity to visualize and localize soft-tissues, which are well-documented to move relative to bony landmarks.^63^ The integration of a soft-tissue imaging system at the exact time of treatment to localize soft-tissue structures including the tumor and organs-at-risk was needed. Advances in volumetric reconstruction and detectors promised to make cone-beam CT a potential solution (see Fig. 9).

### 3.2 Image-Guided Radiotherapy Requirements

The image-guidance problem in radiotherapy had several constraints if it was to be quickly and broadly impactful. From the perspective of quality, images would need to be capable of capturing the contrast between soft-tissues (∼10 to 30 HUs) within the human body and be capable of providing sufficient resolution to localize these and higher-contrast boundaries to within a millimeter in three dimensions. The radiation dose associated with the imaging activity needed to be low enough (∼ few cGy) as to allow multiple imaging events per treatment session and accommodate up to 40 fractions associated with some treatment protocols [e.g., prostate intensity-modulated radiation treatment (IMRT)]. The image needed to be captured with the patient in the position for treatment, such that the image could inform minor corrections in position to assure accurate targeting was achieved before dose delivery. In addition, the field-of-view of the imaging system needed to accommodate the anatomy of interest (up to 50 cm axially and tens of centimeters superior-inferior), including the surrounding organs-at-risk that were to be avoided during the irradiation. Of course, the images needed to be generated in a time interval that would provide confidence that that patient’s anatomy had not moved significantly between imaging, correction, and treatment (<60 to 120 s). This included the capability to take back-to-back images within minutes for verification purposes. In addition to rapid acquisition, the imaging system needed to provide absolute positional information relative to the treatment isocenter and coordinate framework of the treatment machined (sub-mm tolerance for systematic guidance errors). Finally, the imaging system needed to integrate into the existing treatment machines that were increasingly able to generate highly conformal, IMRT plans. Without image-guidance, the improvements in conformality could not be capitalized upon and without image-guidance could put patients at risk. Of course, the current economic paradigm required that the imaging and guidance process could not substantially reduce the overall treatment capacity of the treatment machine if it was to be clinically and operationally viable. Taken together, these constraints represented a substantial challenge that were fortunately well-matched to on-going developments in cone-beam CT reconstruction,^58^^,^^64^ remarkable increases in computational capacity, and progress in the development of large-area, FPIs.^65^

### 3.3 Development of Integrated Cone-Beam CT for Image-Guided RT

The integration of a kilovoltage x-ray sources into a megavoltage radiation therapy treatment machine dates back to the 1950s. These systems were first used to either overcome the poor contrast of the bony anatomy at megavoltage energies^66^ or to overcome the large source size in Co60 treatment machines.^67^ The integration of digital detector systems (key for subsequent volumetric image reconstruction) was first explored by Sephton using a floor-mounted detector in combination with a gantry-mounted x-ray tube.^68^

**Fig. 10:**
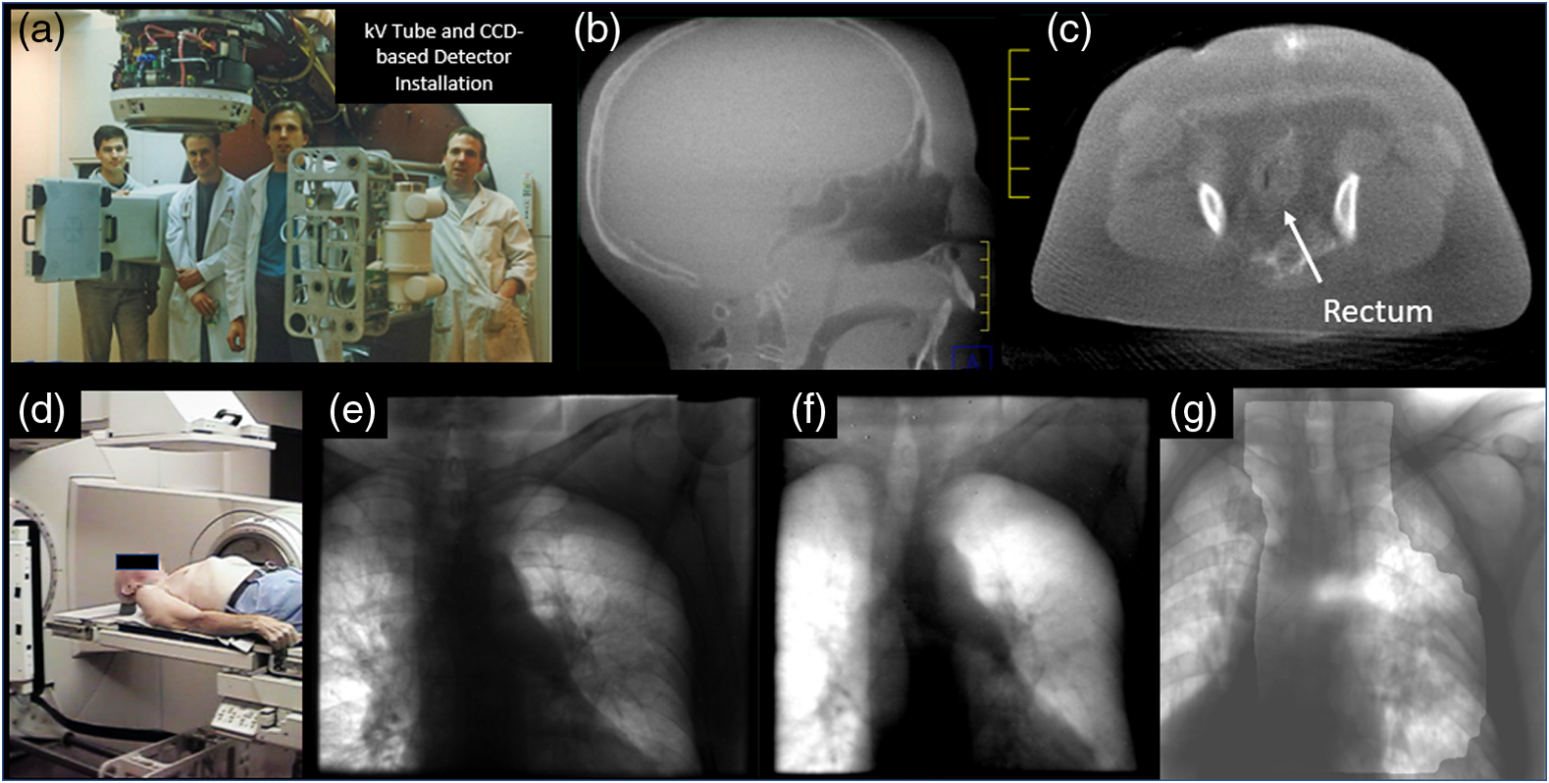
(a) Installation of kV cone-beam CT system on Elekta SL-20 accelerator drum gantry in 1997 (images courtesy of M. Moreau, D. Drake, and D. Jaffray from William Beaumont Hospital and R. Cooke from Elekta). (b) Cone-beam CT sagittal slice through anthropomorphic head phantom. (c) Image of the pelvis of a canine volunteer. (d)–(g) IRB-approved patient study showing kV, MV, and combined radiographic capabilities.

The first fully integrated kilovoltage radiographic and volumetric CT capable radiation treatment device was described in 1997 at the ICCR in Salt Lake City.^69^ This system was built upon a modified Philips SL-20 accelerator (see Fig. 10) that had been adapted to include a retractable x-ray tube and two CCD-based fluoroscopic imaging systems for the orthogonal megavoltage and kilovoltage beams. While clinical utility was promising, many issues related to image quality and geometric accuracy persisted.^70^

**Fig. 11:**
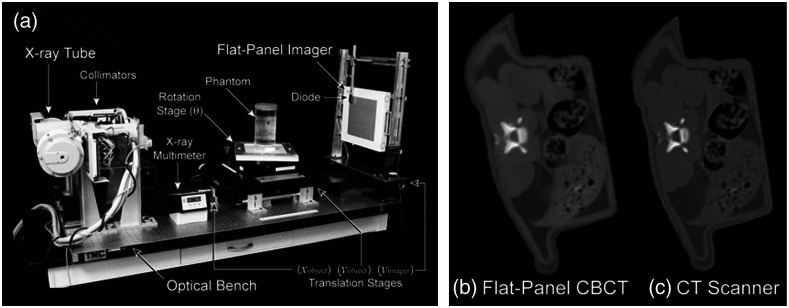
(a) Early work in the evaluation of amorphous silicon FPIs for KV cone-beam CT system in controlled conditions. (b), (c) Axial images of a euthanized rat comparing image quality between flat-panel CBCT and a traditional Philips SR-7000 CT scanner (effective 1 mm slice thickness) suggested soft-tissue imaging was possible with FPIs.

Advances in flat-panel imaging technology were critical for the advancement of geometric stability and for improvements in image quality. Fluoroscopic systems suffered from substantial losses in the optical chain despite the use of high efficiency optics (front surface mirrors, f/0.7 lenses), high efficiency conversion plates (Lanex Fast-Back or CsI:Tl scintillators), and cooled back-thinned CCD detectors.^70^ The development of large area amorphous silicon-based detectors promised to address the coupling issues (Fig. 11) but still suffered from kTC noise, lag, ghosting, and artifacts associated with electronics-related instabilities.^71^^–^^74^ These instabilities were a major draw-back for imaging processes that required highly stable gain and offset corrections for reconstructing volumetric images from projections acquired over ∼60 to 100 s (the rotational gantry speed of a medical linear accelerator).

**Fig. 12:**
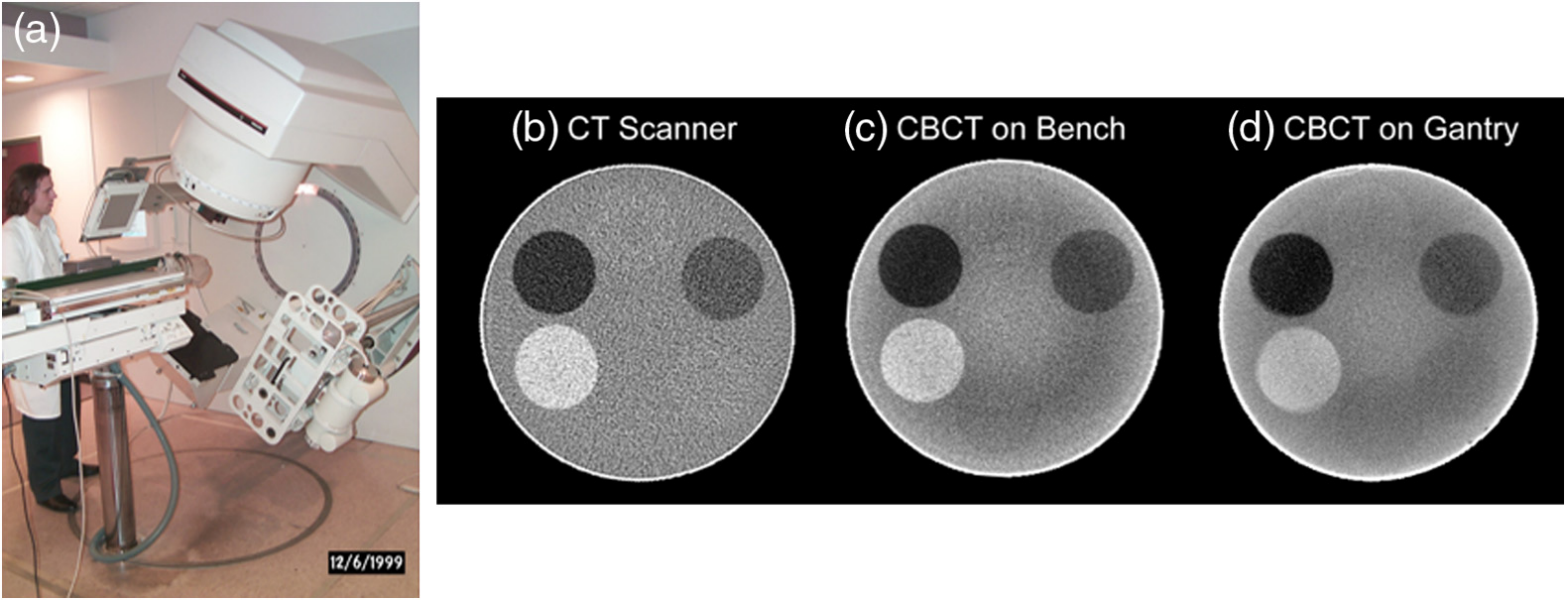
(a) Adaptation of the 20.5-cm FPI to the linear accelerator gantry for studies of image quality and geometric stability. (a)–(c) Comparison of soft-tissue performance between the clinical CT scanner (Philips SR-7000), bench-top, and gantry-mounted systems (1-mm effective slice thickness).

**Fig. 13:**
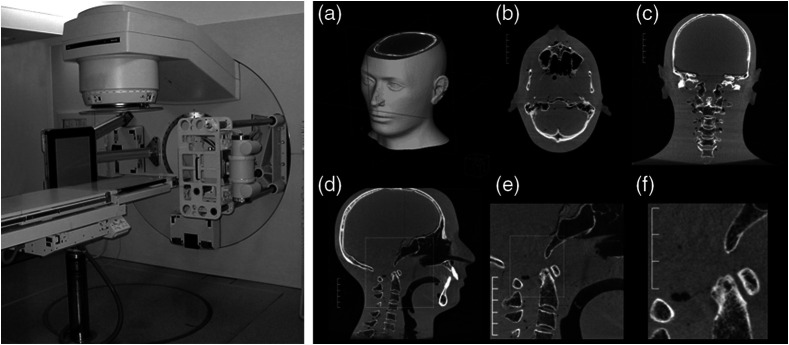
Installation of the large-area 41-cm FPI (Perkin-Elmer RID1640) on the Elekta SL-20 accelerator gantry in 2000. (a)–(d) Reconstruction of anthropomorphic head phantom demonstrating reconstruction in 3D and (d)–(f) remarkable resolution in the progressively zoomed-in sagittal slice set. Reconstructed at 300  μm resolution using modified FDK filtered-backprojection method.

The geometric characteristics of the DFPs were outstanding compared with the lens-mirror-phosphor coupling of the earlier prototypes, making geometric corrections for mechanical instabilities in the overall gantry assembly a tractable problem requiring only routine quality assurance in the clinical setting.^75^ Integrating these corrections into the reconstruction process minimized the need for additional preprocessing.^76^ The resulting images were capable of transferring substantial detail for high contrast objects including fine structure in bony anatomy (see Figs. 12 and 13).

**Fig. 14:**
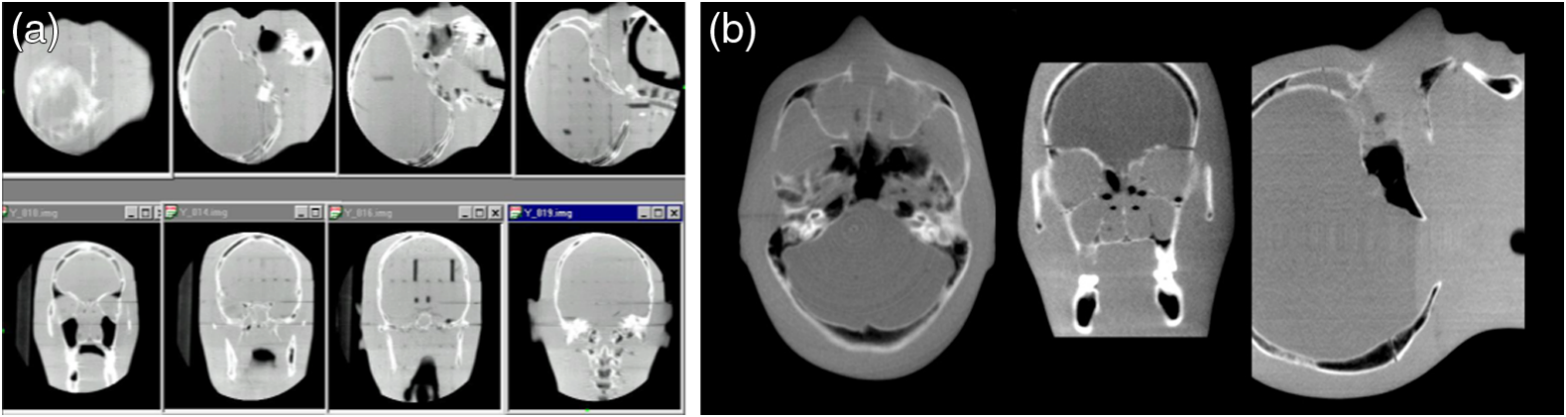
(a) 6-MV conebeam CT using Varian’s a-Si:H portal vision system adapted to a Varian 600CD. Axial and coronal slices demonstrate the advantage of conebeam CT for guidance. The isocenter volume covered is a 20-cm cube, which included nearly the entire head. The total number of monitor units (MU) employed in generating the data set was prohibitive at ∼1500. (b) Axial, coronal, and sagittal CT slices of a head phantom acquired at 6 MV using a 16-cGy irradiation over a 1-min rotation on a Varian 21EX clinac. This was the result of a high efficiency 1-cm-thick pixelated CsI flat panel detector prototype built and tested at MSKCC. It was concluded that this dose is still a factor of ∼3× too high, and the thick scintillator was cost prohibitive for a commercial product.^77^

Efforts to adopt the use of megavoltage beams for cone-beam CT image-guidance were also pursued with reasonable success.^77^^,^^78^ Figure 14 shows the substantial progress made in the development of efficient detectors and flat panel technology. However, the fundamental issues of detector efficiency, treatment collimator limiting field-of-view, and dose to achieve acceptable contract-to-noise prevented commercial success.^79^

Concerns related to the accuracy of simple circular trajectories were also a factor in accepting the utility of cone-beam CT imaging approaches.^80^ More complete analyses of the implications of incomplete data acquisition allowed the community to understand the implications for clinical utility.^81^

Image-quality issues related to the largely unmanaged presence of x-ray scatter generated in the patient reaching the detector continue to be an issue.^82^ Empirical approaches drawn from the traditional CT scanning systems^83^ and measurement-based approaches have been explored.^84^^,^^85^ Several groups continue to develop computational solutions based on analytical and Monte Carlo approaches.^86^^–^^89^ Ongoing efforts on fluence-field modulation approaches offer promise to further mitigate the impact of scatter and better deploy the dose budget for a desired imaging task.^90^^,^^91^ These approaches are applicable to all applications of cone-beam CT.

### 3.4 Clinical Adoption and Ongoing Challenges

**Fig. 15:**
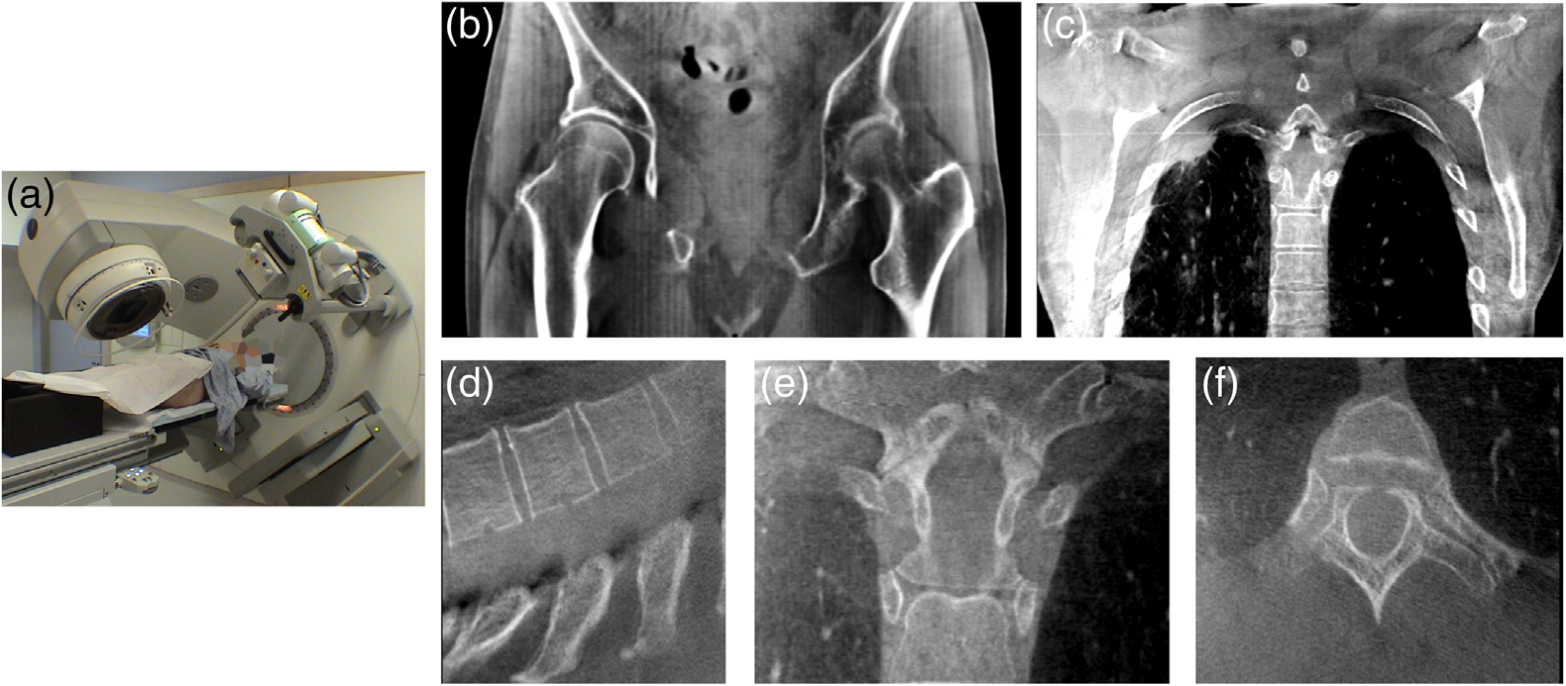
(a) Early prototype of Elekta Synergy at Princess Margaret Cancer Center, Toronto, Canada. (b) Coronal slice showing prostate anatomy. (c) Coronal slice demonstrating lesion in the lung prior to SBRT. (d)–(f) Thoracic spine reconstructed at a resolution of 120  μm. All images acquired with 50-cm diameter FOV using an offset detector, 120 kVp, and <5  cGy imaging dose.

**Fig. 16:**
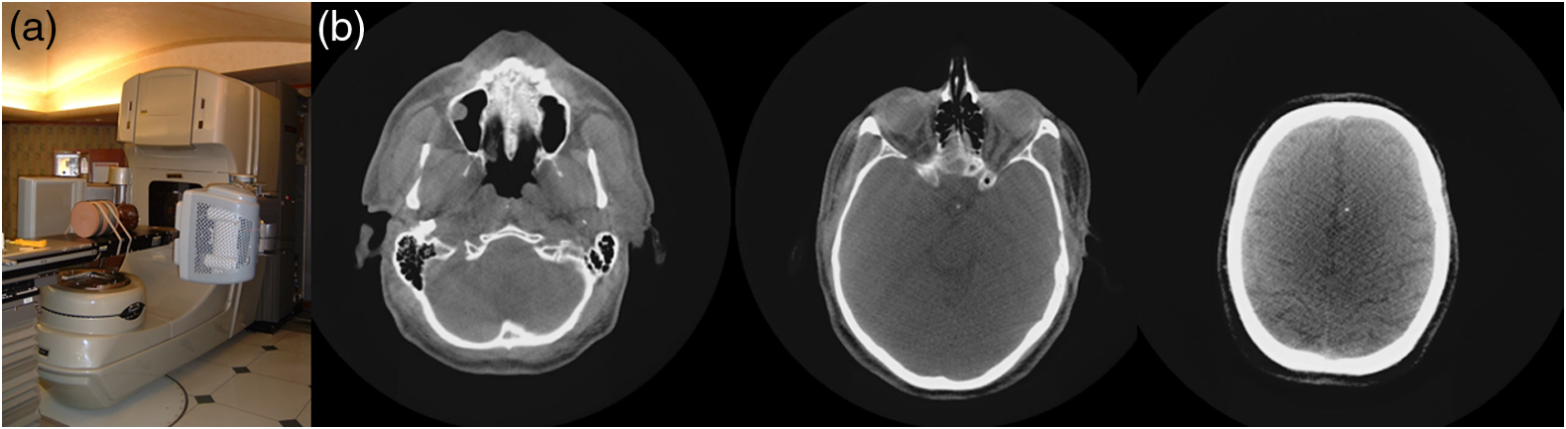
Varian’s on-board imaging system. (a) Geometry on 2100c with robotic arms with imaging phantom. (b) First patient head images dated September 11, 2004, at MSKCC (125 kVp, 80 mA, 8  ms×600 projections, scan diameter 26 cm, axial image 512×512  pixels at 0.5 mm and slice thickness 2.5 mm from five slice average). All images courtesy of Ed Shapiro, Varian.

The clinical utility of these devices depended critically on the development of rapid image reconstruction methods and integrated image-guidance tools to allow on-line images to be used for corrections in position.^92^ Further improvements included the development of four-dimensional (4D) cone-beam reconstruction techniques that enabled projection-based sorting for multiphase reconstructions of the thorax.^93^ These technological advances have allowed image-guidance technologies to be broadly deployed with multiple commercial solution introduced to the market and thousands of these systems deployed in cancer centers around the world (Figs. 15 and 16).

## 4 C-Arm FPCT in Angiography and Image-Guided Interventions

### 4.1 From XRIIs to DFPs: C-Arm Conebeam CT in the Interventional Suite

**Fig. 17:**
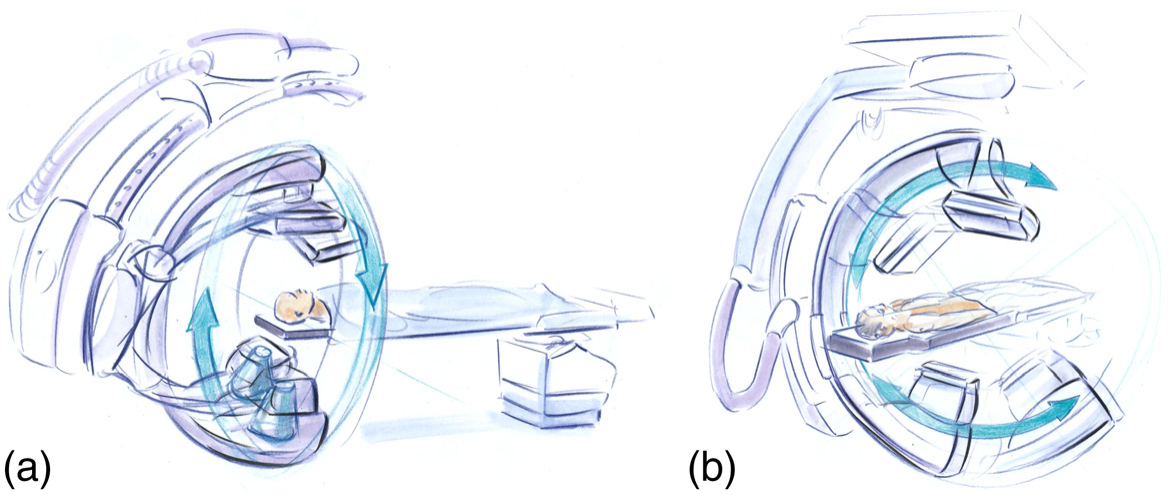
Artist’s sketch of trajectories for conebeam CT acquisitions using a flat-panel C-arm. (a) Propellor mode is used primarily for neurointerventions; (b) patient-side mode is used primarily for body interventional radiology and surgical interventions.

Endovascular therapy options for the treatment of various vascular diseases have exploded since the first interventional coronary treatments. These advances have been made possible mainly through the development of interventional devices and of new techniques for their deployment. In general, image guidance during such procedures is provided by 2D projection images: real-time x-ray fluoroscopy and digital subtraction angiography (DSA). Conebeam CT images provided during an intervention (see Fig. 17) provide benefits for diagnosis, treatment planning, therapy guidance, and outcomes prediction, augmenting the 2D projection images with 3D quantitative information. Conebeam CT is always an adjunct to the main goal of providing real-time, very high-resolution angiograms during subselective injections of contrast. In addition to endovascular therapies, conebeam CT has also proven to be valuable in percutaneous needle-based interventions, such as ablation, biopsies, and vertebroplasty by providing some soft-tissue contrast combined with accurate 3D device and bone visualization to augment real-time 2D x-ray fluoroscopy. 3D imaging capability must be provided without interfering with patient access and standard workflows on C-arm gantries that are used across a wide range of applications from surgical guidance, to neuro interventions, to body and peripheral interventional radiology.

A standard interventional C-arm system has several different operating modes. Real-time low-exposure, high frame-rate fluoroscopy (∼1  μR per frame at the detector, up to 60 fps in cardiology) is used to guide the positioning of endovascular or percutaneous devices. When higher quality diagnostics are needed, a series of high-exposure images (typically two to three frames per second, at 100 to 500  μR per frame at the detector) is obtained. For endovascular interventions, an injection of contrast material (5 to 15 ml) enables vessel visualization. For DSA imaging, a few images without contrast are obtained prior to the injection, and digital subtraction of a “mask”—or noncontrasted—image from the contrast-filled images provides clear visualization of vessels without the presence of background clutter such as bone, airways, or teeth. Fluoroscopy is then used again to guide the placement of devices, embolics, etc., and further high-dose or DSA series are obtained to verify placement of devices and to verify treatment success at the completion of the procedure.

**Fig. 18:**
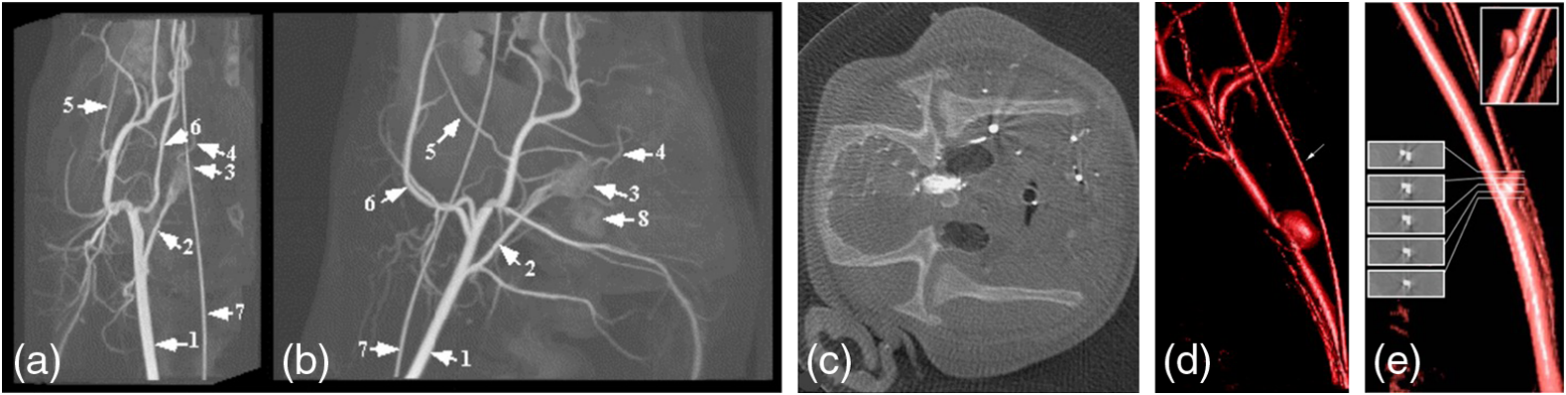
(a) Anteroposterior and (b) lateral maximum intensity projection views through a reconstructed volume of pig cerebral vasculature. Vessel labels: 1, common carotid; 2, ascending pharyngeal; 3, rete; 4, anterior branch of internal carotid; 5, inferior dental; 6, lingual; 7, wire in the endotracheal tube; 8, dense bone surrounding the inner ear. (c) Slice through the 3D volume at the height of the rete; note star pattern surrounding the common carotid due to pulsatile variation of opacification. (d) Surface-rendered image of a 3D reconstruction of a surgically created side-wall aneurysm. (e) In the same aneurysm, a lower injection rate led to incomplete filling of the aneurysm and therefore to underestimation of the aneurysm cross-section.

Proof of principle for C-arm-based conebeam CT imaging was first demonstrated in the 1990s by several different academic-industry partnership teams, using clinically available C-arm systems mounted with XRIIs.^9^^–^^12^ Data were acquired over limited angular range (<60  deg), and algebraic iterative techniques were used to reconstruct 3D vascular trees from few-view (<20) sparse vascular data sets. In London, Ontario, rotational motion of a clinical XRII-based C-arm was tracked using a physical rotary encoder attached to the gantry, angle-dependent XRII distortion was corrected using a 2D grid of beads placed on the XRII,^94^^,^^95^ and the imperfect acquisition geometry (i.e., noncircular) trajectory of the x-ray focal spot was measured (and found to be nominally reproducible) using a single bead fixed in 3D lab space.^96^ Reconstruction using the FDK algorithm with Parker weighting provided some of the first *in vivo* C-arm conebeam CT images of low-contrast structures and contrast-filled vessels, as shown in Fig. 18.^13^

Replacement of the XRII by flat panels enabled acquisition of geometrically accurate, angle-independent projection images with a monotonic signal response that could be corrected to linear. The remaining somewhat unique challenge for C-arm-based CBCT was accurate characterization of the acquisition geometry. 2D images acquired during clinical interventional procedures often require steep angulations, and a C-arm gantry must provide highly flexible positioning. Therefore, the C-gantry is slim and retains some flexibility. However, the acquisition geometry of the mechanical system must be determined to an accuracy that is considerably better than the expected resolution of the final image. An alternative is to compute the trajectory of the source and detector system from a set of image-based 3D-2D correspondences. Using the reconstruction algorithm (and variants) described above, any trajectory that provides sufficient data can then be reconstructed exactly, and approximations can be applied for data acquired using incomplete trajectories. Clinical fixed C-arm systems today rely on reproducible trajectories and use a combination of image-based, external tracker-based (e.g., laser, depth camera) and physics-modeling-based approaches to characterize the intrinsic and extrinsic parameters of the projection matrix for each projection (see Faugeras^95^ for a complete description of projection matrix formalism). Effects, such as wobble due to bearings, and movement of the central ray on the detector due to gravity-induced sag, can significantly degrade the resolution of the CBCT reconstructed volume if not properly characterized or if the system calibration is not up to date. Accurate system calibration is also required to place the reconstructed volume accurately in the angiographic lab space, enabling registration with pre-interventional data sets and accurate comparison of 3D image content pre-, intra-, and postintervention.

Figure 18 shows first *in-vivo* images acquired using the clinical XRII C-arm system in London, Ontario. Image quality is surprisingly good, although several artifacts can be seen, such as streak artifact due to limited number of projection images (only 130) and nonlinear partial volume artifact (further exacerbated by C-arm trajectory wobble, bearing jitter, and sag), beam hardening and time-varying opacification of vessels since imaging was carried during an arterial injection of contrast agent over several cardiac cycles. Other more subtle artifacts include residual veiling glare, and ring artifact due to incomplete relinearization of projection data acquired with a beam-shaping filter (bathtub shape) used to reduce the dynamic range of the images at the detector.

This effect of variable contrast opacification continues to be one of the challenges for FPCT in angiography; the result is two bright streaks per cardiac cycle, radiating out from the center of the vessel and diametrically opposed to each other.^97^ Streaks lead to inaccurate vessel boundaries and may obscure subtle detail and smaller vessels in the tissue surrounding the larger vessel. Nonuniform mixing within an aneurysm may also lead to uncertainty in the aneurysm volume and diameter as shown in Fig. 18.

### 4.2 4D Digital Subtraction Angiography

**Fig. 19:**
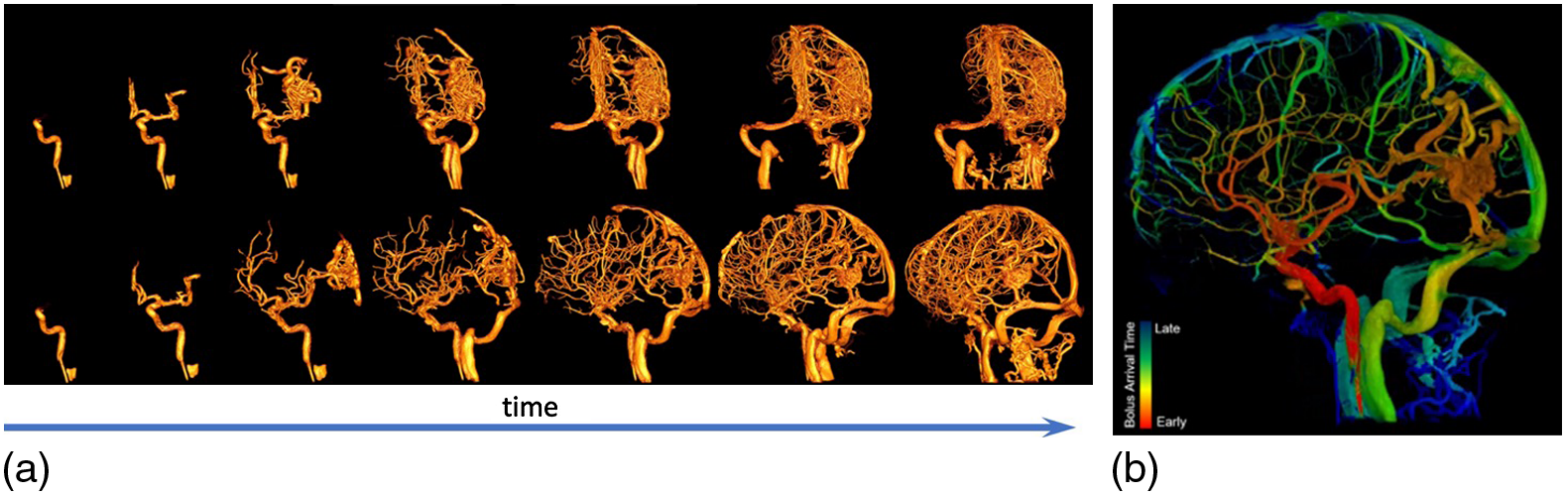
(a) 4D-DSA reconstruction of an arteriovenous malformation. (Top row) Seven 4D-DSA volumes reconstructed from a single extended-arc DSA acquisition viewed from antero-posterior direction; (bottom row) the same volume at the same seven time points as top, but rotated 20 deg between each time point; reconstructed using temporal consistency constraints.^103^ (b) Results from a prototype reconstruction algorithm with estimated bolus arrival time coded in color. All images courtesy of Prof. Charbel Mounayer, Dupuytren University Hospital, Limoges; reconstructions provided by Dr. Annette Birkhold, Siemens Healthcare GmbH.

4D-DSA is an extension of 3D DSA imaging that permits time-resolved visualization of a bolus of contrast through 3D vascular structures. Two different approaches have been proposed. The first from Schmitt et al.^98^^–^^100^ reconstructs a conventional FPCT volume, followed by an additional 2D-digital subtraction angiogram whose dynamic properties are then mapped to the 3D volume. A second approach proposed by Mistretta and coauthors^101^^,^^102^ acquires two “extended arc” mask and fill runs, and a standard 3D DSA volume is reconstructed. A constraint volume is formed by segmenting the vessel tree. Backprojecting each fill-run projection onto the 3D vessel tree results in a volumetric, time-resolved 4D-DSA volume. Example images are shown in Fig. 19. The technique has been successfully applied to visualization of complex vascular disease such as arteriovenous malformations; the ability to see any desired viewing angle at any desired time of bolus passage assists with planning injections of embolic material and has also enabled accurate planning of stereotactic radiosurgery.^104^ Further extensions of the technique, including tracking the time-varying contrast density due to variable dilution over the cardiac cycle, are being explored to provide quantitative estimates of vascular flow.^105^^–^^107^

### 4.3 Motion-Compensated Reconstruction from Single-Sweep Acquisitions

#### 4.3.1 Rigid-motion correction

**Fig. 20:**
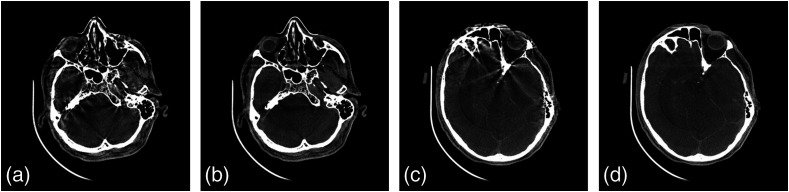
(a) and (c) Motion-corrupted images; (b) and (d) motion-corrected images using epipolar consistency approaches. Clinical images courtesy of Profs. Arnd Doerfler, Stefan Lang, and Philip Hoelter, University Hospital Erlangen and FAU, Erlangen; reconstructions provided by Dr. Michael Manhart, Siemens Healthcare GmbH.

Especially for image-guided neurovascular interventions, correction for rigid motion of the head is key to achieving consistent “close to clinical CT” soft tissue image quality. The challenge is exacerbated by the low frame rates of flat panels, and therefore imaging times can be as long as 10 to 15 s in order to acquire 600 projections. Interestingly, system geometric calibration (discussed above) and motion compensation are closely related, with use of an “objective image quality” metric between simulated and measured projections or iterative correction approaches using image-based metrics such as gradients or entropy to measure artifact levels. An alternative approach exploits redundancies in the projection images, identified via application of epipolar geometry,^26^^,^^108^^,^^109^ and has been applied with success to C-arm CT images of the head, as shown in Fig. 20.

#### 4.3.2 Cardiac motion correction

Single-sweep C-arm CT images can provide good image quality in 3D volumes of the left atrium and pulmonary vein anatomy, as well as the left ventricle.^110^ In addition, adenosine-induced asystole and rapid ventricular pacing have been investigated to improve the image quality of single-sweep acquisitions.^111^^–^^113^ Significant effort has been, and continues to be, dedicated to CT reconstruction of the coronary arteries for guidance of percutaneous coronary interventions as summarized in an excellent review by Cimen et al.^114^ For the coronaries, continuous opacification must be combined with accurate centering of the heart (especially when using a smaller 20  cm×20  cm flat panel) and maintenance of breathhold to limit blurring due to respiratory motion. Tomographic reconstructions methods then apply one of (1) gating, (2) motion compensation, or (3) gating and motion compensation while being adapted to the specific problem of high-contrast moving objects.

#### 4.3.3 Breathing motion correction

**Fig. 21:**
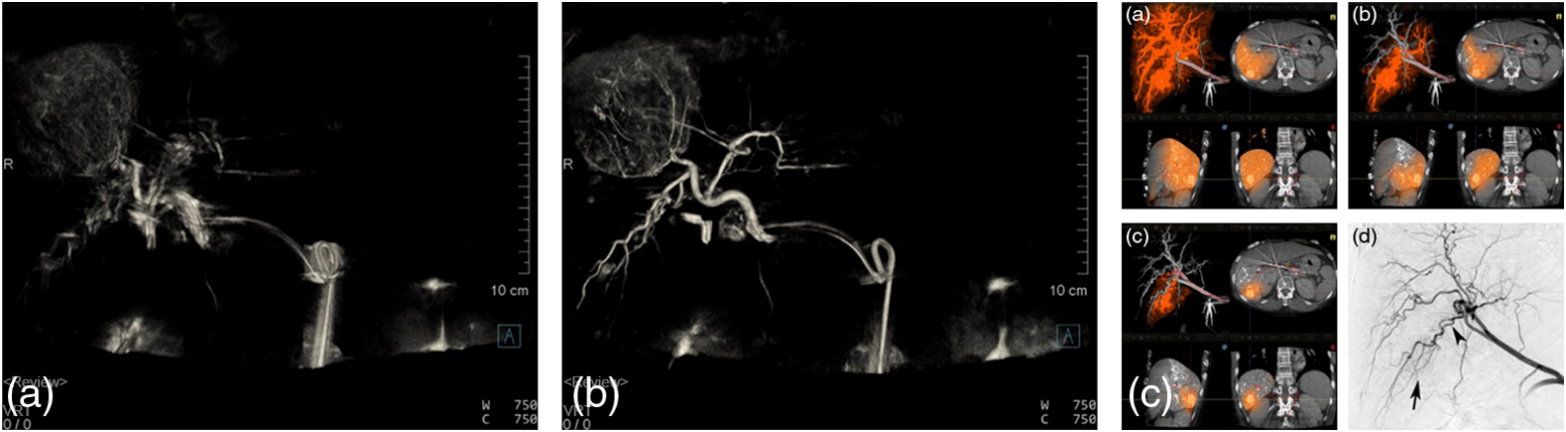
(a) Uncorrected and (b) motion-corrected FPCT reconstruction of an arterial injection of contrast in the liver vasculature including a hepatocellular tumor. Image courtesy of Armeen Mahvash, M.D., MD Anderson Cancer Center, Houston; prototype correction provided by Dr. Guenter Lauritsch, Siemens Healthcare GmbH and Dr. Christopher Rohkohl. (c) Combined use of automated tumor feeder detection and VPP in superselective conventional transcatheter arterial chemoembolization. The virtual embolized area, shown in orange, changed according to the catheter position (an orange marker). The colored area also extended to the right kidney in the axial, coronal, and sagittal directions. (c) Bottom right: common hepatic arteriogram showed a tumor stain (arrow). Reproduced with permission from Ref. 121.

FPCT is used during interventional hepatic vascular procedures such as transarterial chemoembolization (TACE) or selective internal radiation therapy.^115^ Degradation of image quality due to breathing motion has been shown to be a problem especially given the extended breathholds (image acquisition times of 5 to 10 s for 300 projections, corrupt image quality in up to 50% of cases^116^) leading to reduced diagnostic confidence. Using approaches similar to those for reconstruction of moving coronary vessels, reconstruction steps include: (1) reconstruction of a motion-blurred volume; (2) processing of the motion-blurred volume via thresholding, segmentation, landmark identification, etc.; (3) calculation, using a robust cost function, of a nonperiodic, smooth elastic 3D motion vector field describing the nonrigid deformation map between the projection images and the motion-blurred volume; and (4) re-reconstruction of the volume using the motion-corrected projection images. Iterate over steps 2 to 4 using an appropriate stopping criterion^117^^–^^120^ (see Fig. 21 for an example of achievable image quality improvement). Excellent depiction of feeder vessels and tumor vasculature is especially important when image guidance tools such as virtual parenchymal perfusion (VPP) estimation^121^^,^^122^ are used during interventional procedures [see Fig. 21(c)].

### 4.4 Summary of C-Arm FPCT

**Fig. 22:**
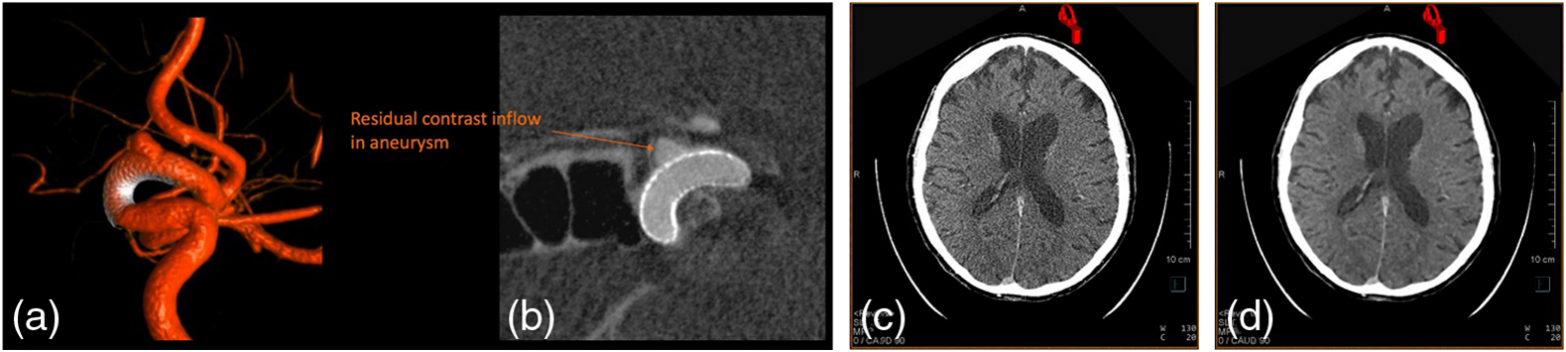
(a) Use of a dual-volume rendering technique to visualize a flow diverter across the neck of an aneurysm; (b) multiplanar reformat image showing the residual contrast inflow in the aneurysm through the flow diverter. Images courtesy David Niemann, MD, University of Wisconsin Hospitals and Clinics Authority. Multiplanar reformat images of a human brain (c) before and (d) after application of a noise-reduction algorithm. Image quality is sufficient to distinguish gray and white matter in the brain, a Hounsfield unit difference of only ∼10  HU.

C-arm-based FPCT continues to evolve beyond the original application of 3D reconstruction of contrast-filled vascular structures (see Fig. 22). As DFP technology and the C-arm gantry hardware evolve, new applications such as multisweep acquisitions for quantitative perfusion imaging in the brain, and multiphase cardiac chamber reconstruction will enter routine clinical use. Although the underlying image quality of C-arm FPCT is approaching that of clinical CT (see Fig. 22), to date no C-arm manufacturer has obtained the FDA label of “computed tomography” for C-arm-based FPCT, preferring to use terms such as “CT-like” to describe the 3D reconstructed data. One can easily predict that as trajectory flexibility, DFP frame rate, noise floor, and stability improve, and new AI-based correction algorithms become standard, it will be possible to achieve the Hounsfield unit stability, uniformity, and reproducibility to merit the label.

## 5 FPCT in Mammography

### 5.1 Breast Cancer Imaging

**Fig. 23:**
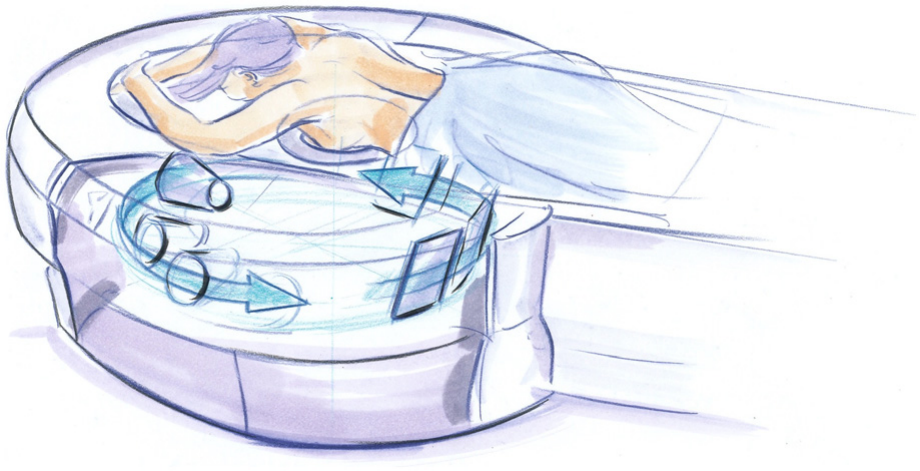
Artist’s sketch of patient setup and tube-detector trajectory for a dedicated breast CT imaging system.

The main motivation for CT imaging of the breast (see Fig. 23) is to eliminate the effect of tissue superposition. In mammography, non contrast-enhanced breast tumors are not differentiated from normal fibroglandular tissue in the breast due to differences in photon attenuation. Rather, x-ray breast imaging is a purely morphological imaging modality. That is, breast tumors are detected because their shape is different from that of normal breast tissue. Therefore, in a 2D modality such as mammography, the overlap of normal tissue projected on top of a pathologic lesion results in a loss of sensitivity.^123^ Furthermore, the overlap of various separate normal fibroglandular strands onto the same location may, randomly, form a shape that mimics a lesion of concern, resulting in a loss of specificity.^124^ To ameliorate this effect, other imaging modalities that decrease the superposition effect have been sought, such as stereoscopic mammography^125^ and digital breast tomosynthesis.^126^^,^^127^ Although the former also showed good performance, the latter has been widely introduced for every-day clinical breast imaging.^128^ However, even though tomosynthesis does provide some information in the third dimension, its resolution in this direction is still quite limited.

### 5.2 Breast Imaging Requirements

**Fig. 24:**
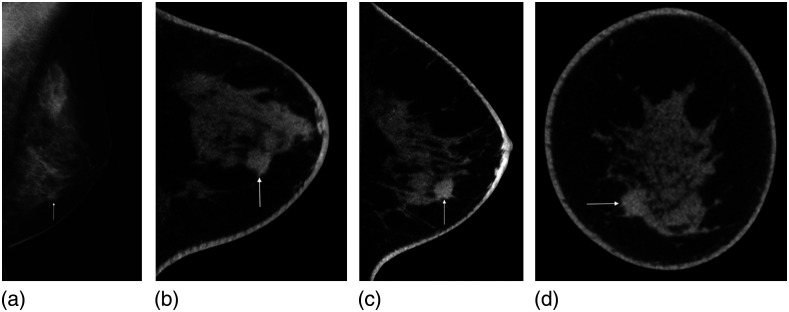
(a) Mediolateral oblique view mammogram depicting a ductal invasive carcinoma as a suspicious mass in the lower part of the breast (arrow). (b) Axial, (c) sagittal, and (d) coronal cone-beam breast CT images with a highly suspicious mass (arrows). Reprinted from Wienbeck et al.^129^ with permission from Elsevier.

Breast cancer detection and diagnosis is an especially demanding clinical application for imaging technology due to the need to adequately image two very different types of lesions: soft tissue-based and microcalcifications.^130^ The former usually presents as masses with or without spiculations, in addition to some other forms, such as architectural distortions. These lesion types are of low contrast and in the mm to cm scale (Fig. 24). Therefore, low energy x-ray spectra are needed to increase subject contrast, whereas spatial resolution is not at a premium (with the caveat that mass edge characterization and detection of spiculations, important for diagnosis, do benefit from high spatial resolution) compared with body imaging. However, the calcifications can be as small as 200  μm, resulting in the need for high spatial resolution. Their small size also makes calcification detection quantum-noise limited,^131^ resulting in the need for higher doses.

Therefore, a conebeam CT system for breast imaging needs to balance various competing requirements. The use of softer x-ray spectra to increase contrast, for improved mass visibility, results in reduced transmission, decreasing dose at the detector, increasing noise, and hence decreasing calcification visibility. To compensate for this, increases in tube current and/or x-ray pulse length are needed, but of course these increase the dose to the breast. Furthermore, increasing the pulse length also results in an increase in the effective focal spot size, reducing the spatial resolution of the system.

In addition, as opposed to all other flat-panel conebeam CT uses, the main application in breast imaging would ideally be screening of the general population. This, of course, makes radiation dose a real concern, realistically limiting it to a level similar to that currently used for 2D mammography-based screening.

Although the use of iodinated contrast enhancement in breast imaging results in substantially improved performance,^132^ its use in screening of the general population and for first-line diagnostic work-up is not clinically feasible. Therefore, the contrast demands of this clinical application cannot be ameliorated by the use of iodinated contrast.

On the other hand, except for specific cases, which right now are only in the research realm, breast imaging does not require fast acquisitions. In fact, conebeam breast CT acquisitions are currently 10 to 16 s long.^133^^,^^134^ In a study involving 40 patient conebeam breast CT acquisitions with a system with a 10-s acquisition time, only two of the cases (5%) were found to suffer of motion artifacts due to the relatively long acquisition time.^133^

### 5.3 Flat-Panel Conebeam Breast CT Systems

The most obvious aspect that differentiates conebeam breast CT from the rest is its geometry. Strictly speaking, breast CT uses a half-cone beam geometry, with the central ray located at one edge of the x-ray beam, to graze the chest wall of the patient being imaged. To maximize tissue coverage, the size of the inactive area of the flat-panel detector edge on the chest wall side needs to be minimized.

**Fig. 25:**
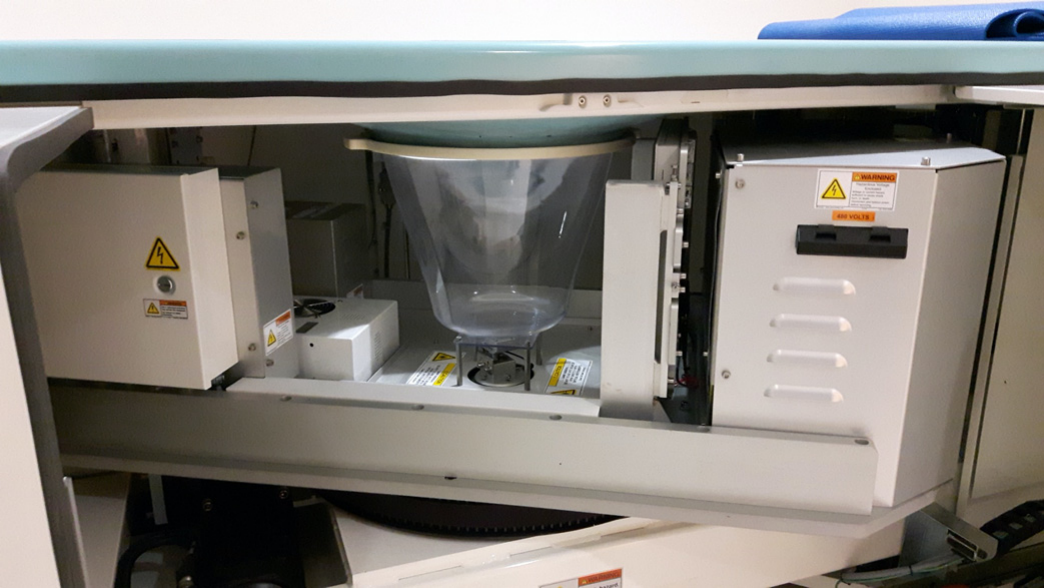
Photo of a flat panel cone beam dedicated breast CT system showing the main components of the imaging chain. The plastic cup located surrounding the space for the pendant breast isolates the moving parts of the system from the breast and the rest of the patient.

This geometry also results in breast CT involving a maximum cone-beam angle that is larger than those of other applications. To improve on the resulting limitations in undersampling, alternative acquisition geometries have been investigated. In one breast CT implementation, a saddle trajectory was proposed, resulting in improved coverage and sampling of the frequency space.^135^ In more recent work, implementations involving multiple x-ray sources have been investigated using computer simulations.^136^ Other image acquisition trajectories, such a full revolution plus a linear scan, have been previously proposed.^137^ However, to date, all current flat-panel-based breast CT implementations that have reached the stage of patient imaging, either as commercial or research systems, use the traditional half-cone beam geometry with a single circular scan with one x-ray source (Fig. 25).

Two types of DFPs have been used in flat-panel conebeam breast CT systems. The initial breast CT systems rely on a-Si with a columnar Csi:Tl scintillator-based TFT DFP. The usual size of these DFPs is 40  cm×30  cm, which are large enough, considering the magnification factors of breast CT systems, to allow for the imaging of a whole breast in one projection.^133^^–^^135^^,^^138^^,^^129^ Some more recent system designs have incorporated the use of CMOS-based DFPs, which tend to be smaller in size, in the order of 29  cm×23  cm. The use of this new DFP technology has allowed for an increase in spatial resolution due to their smaller pixel pitch (Table 1).

Some early implementations of breast CT systems did not involve pulsed x-ray sources, of course impacting spatial resolution. However, current systems all use pulsed sources, which, together with the transition to CMOS-based detectors, have resulted in substantially improved spatial resolution in breast CT.^139^ As is shown by Gazi et al., increasing tube current does not seem to result in a substantial impact in the focal spot size.^139^ This is important since increasing the tube current allows for maintaining a short pulse length, crucial to minimizing the effective focal spot size in the direction of tube travel.

It should be noted that a completely different breast CT system design involves spiral imaging with an x-ray fan-beam and the use of a strip photon-counting detector.^140^ However, since this is not a cone-beam CT system, it will not be discussed further.

### 5.4 Scatter Correction

**Fig. 26:**
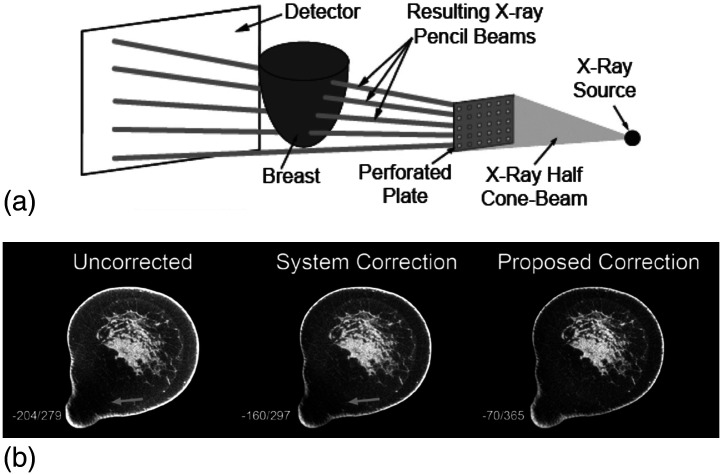
(a) Schematic of the beam pass array method, which results in a sampling of the primary-only signal in the FPCB dedicated breast CT projection. (b) Correction of the acquired projections results in improved contrast and increased homogeneity throughout the reconstructed image. The projection-domain correction using information acquired for the specific imaged breast yields superior results compared to the image-domain correction performed by the system. Reprinted from Sechopoulos^126^ with permission from AAPM, and from Ramamurthy et al.^143^ © Institute of Physics and Engineering in Medicine, reproduced by permission of IOP Publishing, all rights reserved.

Various different approaches for x-ray scatter correction have been proposed for flat-panel conebeam breast CT. Of course, one approach, which is, strictly speaking, not correcting for scatter, is to correct for the resulting cupping artifact postreconstruction.^141^^,^^142^ One method that has been proposed to estimate the scatter signal specific for the imaged breast is the beam pass array method (Fig. 26).^143^^–^^145^ One important benefit of this method is that although it requires the acquisition of additional images of the breast, the presence of a beam pass array between the source and breast substantially reduces the dose of these extraprojections. The inverse of this approach, the beam stop array method, has also been proposed for breast CT.^146^ To limit the increase in dose involved due to the acquisition of extraprojections for the beam stop array method, only a few projections are acquired and the rest of the information is estimated via interpolation.

Other computational approaches, which do not require the acquisition of additional projections of the breast in question, have been also proposed.^147^^,^^148^ Currently, the single commercial FDA-approved conebeam breast CT system incorporates a cupping artifact correction postreconstruction only. Therefore, the commercial suitability of all the other methods that involve scatter correction is not yet clear.

### 5.5 Contrast-Enhanced Breast Imaging

**Fig. 27:**
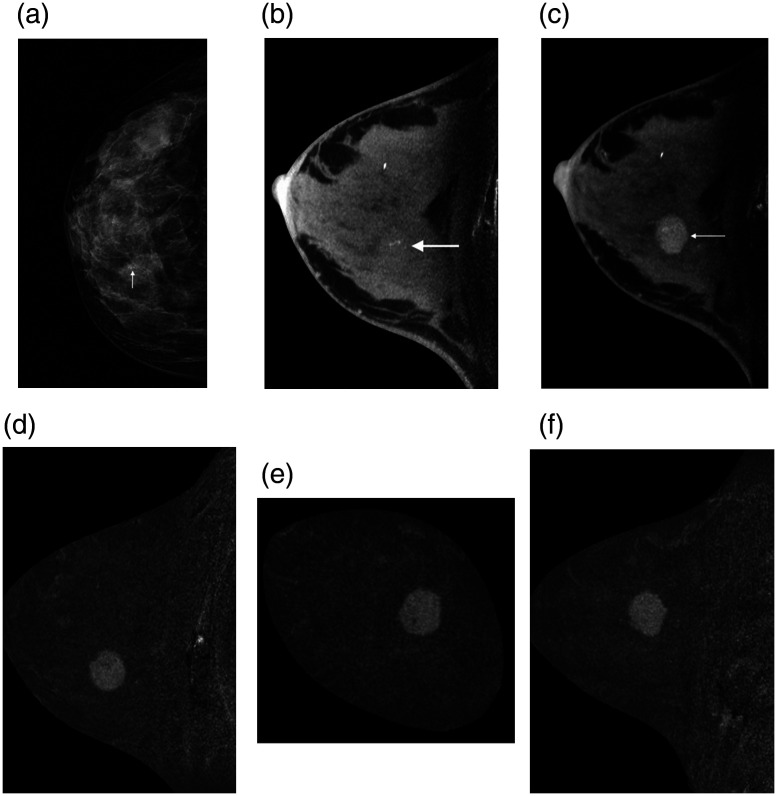
(a) Craniocaudal (CC) view mammogram showing suspicious clustered microcalcifications (arrow) in the inner part of the right breast. The density of the tumor is superimposed by normal breast tissue. (b) The microcalcification cluster is visible in the dedicated noncontrast cone-beam breast CT (arrow). (c) Addition of contrast for contrast-enhanced cone-beam breast CT shows a clearly delineated, intensely homogeneous contrast enhanced tumor mass (arrow). (d) Axial, (e) coronal, and (f) sagittal views of the subtraction images show the enhancement in the tumor mass, which was determined to be a ductal invasive carcinoma. Reprinted from Wienbeck et al.^129^ with permission from Elsevier.

The use of iodinated contrast for breast cancer imaging allows for the interrogation of the vascular status of the breast and, specifically, that of suspicious lesions. The rapid angiogenesis promoted by signaling from the tumor to allow it to grow results in malformed, leaky, blood vessels. When iodinated contrast is injected intravenously, the iodinated blood mixture circulates and leaks out of these tumor vessels. Contrast-enhanced breast imaging, therefore, aims to image this extravasated iodinated blood, resulting in images in which malignancies have considerably higher contrast (Fig. 27).

The x-ray spectra used in conebeam breast CT systems, of considerably lower tube voltages than those in other CT applications, are ideally suited for contrast-enhanced imaging, given the closeness of the energy of the x-rays to the k-edge of iodine.^149^ Therefore, there are multiple studies that have shown the improved performance of contrast-enhanced imaging for this application.^138^^,^^150^^,^^151^

One particular advantage that makes breast CT amenable to contrast-enhanced imaging is that, as opposed to in mammography and tomosynthesis, no breast compression is used. Therefore, there is no restriction of blood flow into the breast during acquisition, as with those modalities. This makes image acquisition with short delay after injection and/or of multiple images postinjection much easier to implement.

### 5.6 Dynamic Perfusion Breast Imaging

Due to this possibility of performing dynamic imaging, among other advantages, it has been proposed recently that flat-panel conebeam breast CT may be ideally suited for dynamic perfusion imaging. An optimized implementation of dynamic CT perfusion for breast cancer imaging would result not only in iodine-only images in which lesions are better depicted but also would result in the acquisition of enhanced functional information. Incorporation of high-speed added filtration switching,^152^ spectral reconstruction,^153^^,^^154^ and motion correction^155^ could yield dynamic images with very high combined spatiotemporal resolution. These additional capabilities could provide valuable information to, especially, determine tumor status and help predict tumor response to neoadjuvant therapy. As opposed to current flat-panel conebeam breast CT systems, such a system should be aimed to have a reduced acquisition time, in addition to the ability to acquire multiple images with minimal or no delay in between acquisitions.

## 6 Dedicated Head and Extremities Flat-Panel CBCT

### 6.1 Introduction to Dedicated Head and Extremities FPCT

**Fig. 28:**
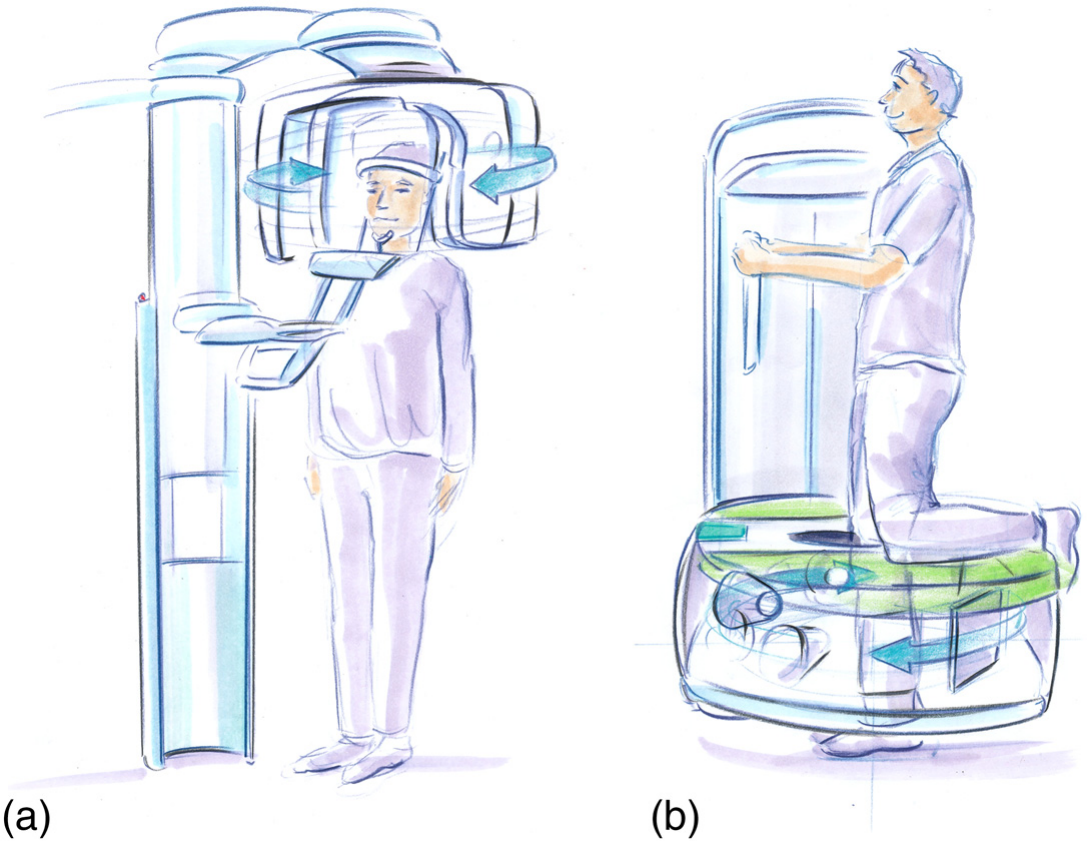
Artist’s sketch of (a) a dedicated FPCT system for head imaging and (b) an extremity FPCT imaging system in the “weightbearing imaging” position.

Flat-panel-based CBCT for dedicated head and extremities systems was born out of contemporaneous developments in imaging for radiotherapy and interventional imaging (Fig. 28). Like interventional CBCT, the first 3D systems for dental and maxillofacial imaging were based on image intensifiers.^8^^,^^156^^–^^158^^,^^159^Initial FPCT systems for dental applications were reported in the early 2000s by Sukovic et al.^160^ and Baba et al.^161^ These advances were quickly followed by a large number of successful commercial FPCT offerings. Farman and Scarf^162^ and Molteni^163^ each have a detailed history and summary of FPCT head imaging systems. The success and development of dedicated CBCT continue to broaden and extend to other applications including extremities imaging.

One of the reasons for the success of these dedicated systems is the ability to customize the device to the anatomical and clinical target. For example, by focusing on a single clinical target (e.g., head or extremities) as opposed to general full-body CT, a number of optimizations can be made that can have a distinct advantage over traditional diagnostic CT.

### 6.2 Customization of Flat-Panel CBCT for Head Imaging

There are a number of factors that have contributed to the success of flat-panel CT for dedicated head imaging. These factors were particularly well-aligned for dental imaging with dedicated CBCT being described by some as the “most significant advance in dental imaging since the introduction of rotational panoramic radiography.”^162^ The matching of the flat-panel technology to the particular clinical application and environment contributed to the early adoption of flat-panel CBCT for dental imaging. That success, in turn, has enabled other dedicated head imaging applications such as temporal bone and sinus imaging for otolaryngology.

#### 6.2.1 High-resolution, 3D capability

The high-resolution capability provided by flat-panel detectors is particularly important in dental, maxillofacial, and temporal bone imaging. Visualization of submillimeter anatomical features—e.g., in and around teeth in the mandible and maxilla, of the inner ear bones and semicircular canals, and air–tissue–bone boundaries in the paranasal sinuses—is critical for the diagnosis and treatment of a range of conditions. At the advent of flat-panel CT, traditional diagnostic multirow detector CT typically produced images with anisotropic resolution with a slice thickness greater than the axial, in-plane resolution. Flat-panel CBCT with its isotropic submillimeter resolution permitted not only better visualization of small features but also the ability to produce high-quality oblique slices and curved planar reformations to highlight specific anatomy (e.g., double oblique reformatting to visualize the stapes in the inner ear^164^ and curved cross-sections along the dental arch).^165^ In dental imaging, which was previously largely focused on 2D projection imaging, the capacity for 3D assessment and quantitation was a distinct advantage.

#### 6.2.2 Compact system design

Traditional multirow CT scanners are large devices with complex siting requirements including significant requirements for electrical power, structural and load-bearing capacity, and radiation shielding. These elements are an obstacle to point-of-care imaging in dental offices (where x-ray imaging was common but with much smaller devices) and private practice physicians’ offices (where imaging was often conducted out-of-office). By focusing on head imaging alone, a number of physical aspects of the device may be optimized leading to a compact design.

**Fig. 29:**
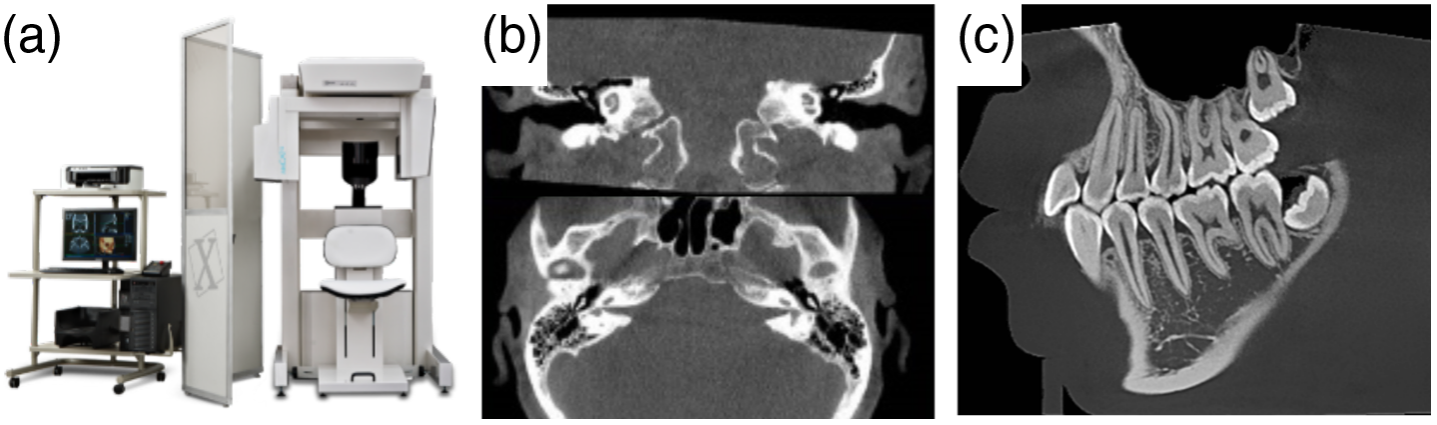
(a) Illustration of a dedicated head CT scanner for otolaryngology applications. The seated design is compact and requires only modest shielding requirements. (b) The high-contrast, high-spatial resolution is excellent for visualizing the temporal bone and inner ear structures. (c) Another sample image from a dedicated dental scanner showing interfaces between teeth and bone as well as position within the mandible and maxilla. Panels (a) and (b) are courtesy Xoran Technologies, Inc.; panel (c) is courtesy Yuxiang Xing, Tsinghua University.

Flat-panel detectors are themselves are relatively thin and lightweight. Their large area permits wide coverage in a single circular orbit, covering the mandible and maxilla, paranasal sinuses, or the temporal bones relatively easily. There is much less variability in patient head size as compared with abdominal imaging, and the sizes are smaller permitting smaller source-to-detector distance in a dedicated system. Combined with the focus on relatively high-contrast applications (e.g., bone quality and structure, soft-tissue/bone interfaces in dental imaging, and soft-tissue/bone/air boundaries in sinus and inner ear imaging), there are also much lower power requirements on x-ray tube power. While care must be taken to balance focal spot size effects (important for high resolution scanning) with the short source-to-detector distance,^166^ the modest x-ray power needed generally allows for the use of compact “monoblock” designs. Such x-ray sources have all of the high voltage electronics within the same structure as the x-ray tube eliminating the need for bulky high-voltage cabling and reducing the relative power requirements. Combined with the modest rotation speeds that require smaller, lower power motors, most dedicated head scanners can be powered off standard electrical outlets. The form factor of head scanners is also optimized—often for scanning in a standing or seated position. This small footprint is a distinct advantage for placement at the point-of-care in a physician’s office. An example dedicated cone-beam CT head scanner is shown in Fig. 29.

#### 6.2.3 Low cost and low dose

Another important element of dedicated head scanners is the relatively low cost as compared with traditional multirow CT. Much of the cost reduction goes hand-in-hand with the compact design, modest gantry requirements (i.e., smaller x-ray tubes, and smaller motors), as well as the relatively inexpensive flat-panel detector. It is widely reported that flat-panel CBCT dose is lower than conventional CT.^167^^–^^169^ This is, in part, by design with the focus on high-contrast features and low tube power, which limits the radiation exposures that are available. However, there are also geometric advantages as compared with diagnostic CT (e.g., no overshoot due to neighboring “slices” that one would find in a spiral acquisition). The relatively low exposures contribute additionally to lower cost of these systems with modest siting requirements including simpler shielding plans.

### 6.3 Clinical Applications for CBCT Head Imaging

**Fig. 30:**
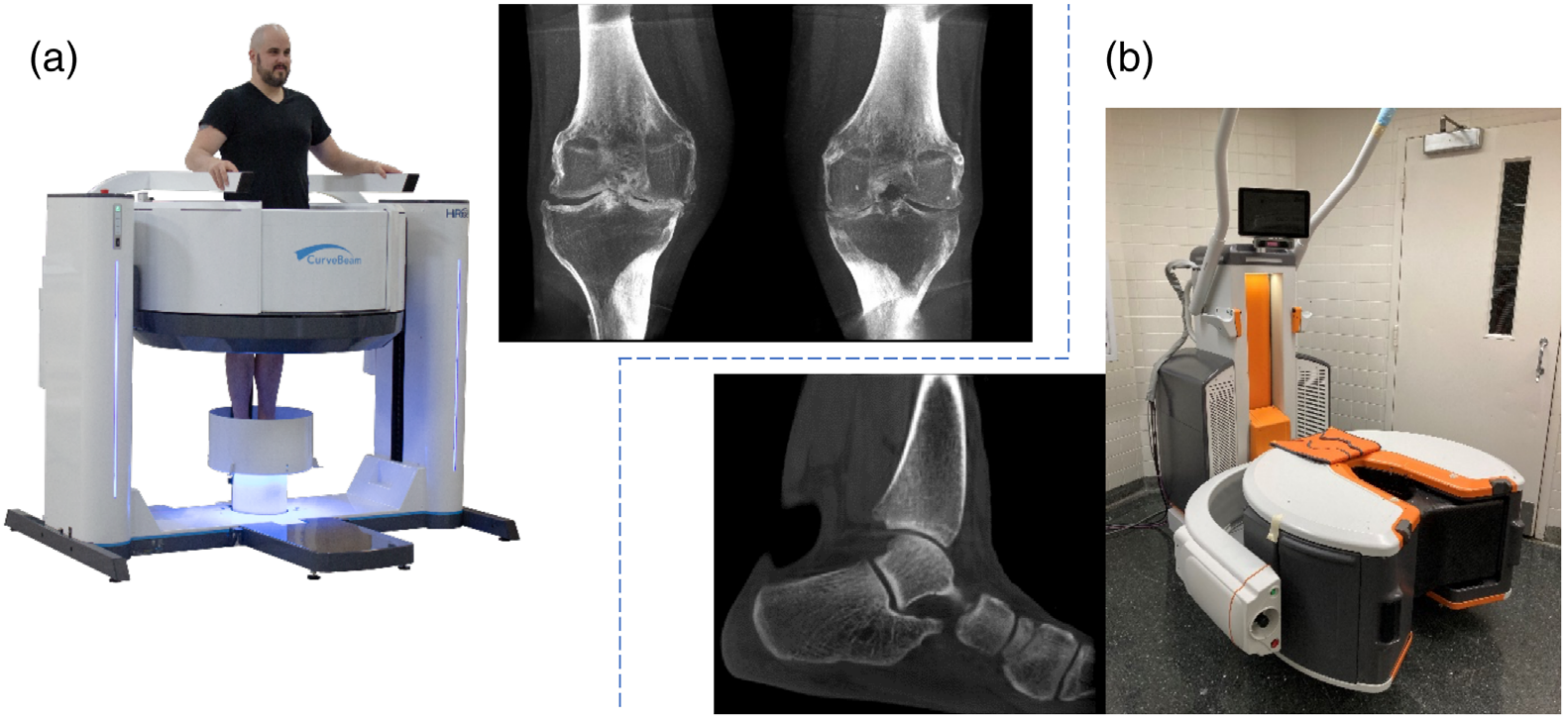
(a) Illustration of a dedicated extremities CBCT capable of bilateral leg scans. The weight bearing scan shows reduced joint space in a sample knee scan. (Images courtesy CurveBeam). (b) Another extremities CBCT that targets individual extremities with a short scan orbit and detector that passes between the legs. A sample ankle image shows the relation between bones and surfaces in this complex joint. (Images courtesy Wojciech Zbijewski, Johns Hopkins University.).

There is a plethora of clinical imaging applications reported in the literature.^170^ Initial application of dental scanners focused on oral surgery planning^158^ and implant planning^171^^–^^173^ but has grown to include evaluations of implant failure.^174^ Critical elements of these studies include assessment of bone quality including height and width (e.g., of the mandible)^175^ and bone mineral density,^176^ 3D assessment of alveolar ridge, identification of vital structures including the inferior alveolar nerve, and modeling for the fabrication of surgical guides.^175^ (Sample image is shown in Fig. 30.) Other applications include the evaluation of mandibular fractures,^176^ the temporomandibular joint,^177^^,^^178^ and tooth root fractures.^179^ Otolaryngological applications include assessments of inner ear disease and the temporal bone^180^ (see Fig. 30) including assessments of semicircular canal dehiscence,^181^ otosclerosis,^182^ and cochlear implant placement.^183^^–^^185^ Dedicated head CBCT has also found application in diagnosing and gauging inflammatory sinusitis^186^^,^^187^ as well as in the planning and assessment of sinus^188^ and maxillofacial surgeries.^189^

### 6.4 Customization of Flat-Panel System for Extremities Imaging

Many of the customizations for head scanning apply to extremities scanning as well. In particular, the focus on high-resolution targets including bone structure, morphology, and interfaces is well-suited to flat-panel CBCT. Again, focusing on extremities targets that are smaller than general, full-body, multirow CT requirements permits compact designs and significant cost savings. Many dedicated extremities system integrate self-shielding into the scanner design with lead drapes, etc. to further reduce siting requirements. As with head scanning, dedicated extremities generally operate at lower dose^190^^,^^191^ than multirow CT, further reducing siting requirements.

#### 6.4.1 Weight-bearing imaging

One element that is distinct for extremities imaging is the customization of scanners for weight bearing^192^^–^^194^ imaging. In contrast to multirow CT where patients are lying flat on a scanning table, dedicated extremities CBCT have been designed to permit scanning of standing patients. Imaging of patients in natural poses and of anatomy in the loaded state allows visualization of joint spaces, impingements, erosions, etc. under weight-bearing conditions.

Examples of two extremities scanners are shown in Figs. 29 and 30, where design choices have been made to scan either both legs or single legs, respectively. Note that accessibility is particularly important for patients with joint and mobility issues, and these systems are designed with additional arm supports.

### 6.5 Clinical Applications for Extremities Imaging

Dedicated extremities CBCT have been used to diagnose a wide range of issues including arthrography,^195^ fracture assessment,^196^^,^^197^ and fracture healing.^198^^,^^199^ There are ongoing studies looking into the efficacy of dedicated systems for the evaluations of bone health including measures of bone mineral density^200^ and assessment of osteoporosis and osteoarthritis.^201^^,^^202^

### 6.6 Point-of-Care Dedicated CBCT

One of the main advantages of dedicated CBCT is the ability to perform point-of-care scanning. This has the potential to streamline patient care and have diagnostic imaging, diagnosis, and treatment in one center. The low-cost and compact form factors of the CBCT systems allow these devices to be put in smaller institutions and individual private practices providing much convenience for the patient. However, such installations raise additional challenges including the potential for overuse through self-referral and the need for robust quality assurance. Trained and qualified medical physicists and engineers need to be part of a regular maintenance program to ensure proper quality control in image quality, radiation dose, protocols, shielding, etc.

### 6.7 Future of Dedicated CBCT Imaging

Flat-panel CBCT is finding its way into other dedicated applications such as spine imaging,^203^ which overlaps somewhat with other interventional systems described previously in this paper. Some applications of flat-panel CBCT have been driven by advances in soft-tissue image quality. For example, through rigorous modeling of the many nonidealities that flat-panel systems present (including detector lag, detector glare, and scattered radiation), sophisticated correction schemes have been developed^204^ that permit good soft tissue visualization. Recent studies^205^ have shown that dedicated CBCT image quality can be sufficient for intracranial diagnosis and monitoring including hemorrhage and hydrocephalus. Mobile dedicated systems permit point-of-care deployment in critical/intensive care units and can potentially be brought to the patient bedside, eliminating the need to transport critically ill patients between departments in the hospital.

## 7 Future Enabled by New DFP Developments

Flat-panel CT has become an integral component of diagnostic imaging, radiation therapy image guidance, and image-guided interventional therapy. As DFPs have evolved, so has the image content of the 3D images and therefore developments in DFP technology point to future opportunities in FPCT. A first area of innovation is the construction of dual-layer dual energy flat panels. Feasibility has been demonstrated using the DL DFP, which consists of a top layer with a 200-μm-thick CsI scintillator coupled to an amorphous silicon (aSi) DFP of 150-μm pixel size and a bottom layer with a 550-μm-thick CsI scintillator coupled to an identical aSi DFP. The two layers are separated by a 1-mm Cu filter to increase spectral separation. Applications such as dual-energy CBCT for contrast enhancement and material decomposition have been demonstrated. The merits of the DL detector include superior spatial and temporal registration between its constituent images and less complicated acquisition sequences as compared with a kV-switching approach.^206^^,^^207^ In the context of the Horizon 2020 EU funded activities, a consortium has come together to investigate the use of organic photodetectors (OPDs) as a transparent substrate, for use in multilayer indirect-conversion DFPs. Visible-light OPDs offer cost-effective fabrication methods using low temperature processes, making them particularly attractive for large area image detectors on lightweight flexible plastic substrates.^208^^,^^209^ Direct-conversion photon-counting energy discrimination detectors have also been investigated in the context of FPCT spectral imaging, and the results are promising.^210^^–^^212^ First results from a hybrid aSi:H/direct-conversion strip detector have been evaluated in the context of interventional neurology, demonstrating the possibility for energy-based discrimination between intracranial hemorrhage, calcifications, and iodine staining from periprocedural contrast-enhanced imaging sequences.^213^
